# Senicapoc treatment in COVID‐19 patients with severe respiratory insufficiency—A randomized, open‐label, phase II trial

**DOI:** 10.1111/aas.14072

**Published:** 2022-05-13

**Authors:** Asger Granfeldt, Lars W. Andersen, Mikael F. Vallentin, Ole Hilberg, Jørgen B. Hasselstrøm, Lambert K. Sørensen, Susie Mogensen, Steffen Christensen, Anders M. Grejs, Bodil S. Rasmussen, Klaus T. Kristiansen, Thomas Strøm, Isik S. Johansen, Olav L. Schjørring, Ulf Simonsen

**Affiliations:** ^1^ Department of Anesthesiology and Intensive Care Aarhus University Hospital Aarhus Denmark; ^2^ Department of Clinical Medicine Aarhus University Aarhus Denmark; ^3^ Prehospital Emergency Medical Services Central Denmark Region Denmark; ^4^ Research Center for Emergency Medicine Aarhus University Hospital Aarhus Denmark; ^5^ Department of Medicine Vejle Hospital Vejle Denmark; ^6^ Section for Forensic Chemistry, Department of Forensic Medicine Aarhus University Aarhus Denmark; ^7^ Department of Biomedicine, Pulmonary and Cardiovascular Pharmacology Aarhus University Aarhus Denmark; ^8^ Department of Anesthesia and Intensive Care Aalborg University Hospital Aalborg Denmark; ^9^ Department of Clinical Medicine Aalborg University Aalborg Denmark; ^10^ Department of Anesthesiology Hvidovre Hospital Hvidovre Denmark; ^11^ Department of Anesthesiology Odense University Hospital Odense Denmark; ^12^ Department of Anesthesiology, Hospital of Southern Jutland University of Southern Denmark Odense Denmark; ^13^ Department of Infectious Diseases Odense University Hospital Odense Denmark

**Keywords:** ARDS, COVID‐19, respiratory insufficiency, SARS‐CoV‐2, Senicapoc

## Abstract

**Background:**

The aim of the current study was to determine if treatment with senicapoc, improves the PaO_2_/FiO_2_ ratio in patients with COVID‐19 and severe respiratory insufficiency.

**Methods:**

Investigator‐initiated, randomized, open‐label, phase II trial in four intensive care units (ICU) in Denmark. We included patients aged ≥18 years and admitted to an ICU with severe respiratory insufficiency due to COVID‐19. The intervention consisted of 50 mg enteral senicapoc administered as soon as possible after randomization and again after 24 h. Patients in the control group received standard care only. The primary outcome was the PaO_2_/FiO_2_ ratio at 72 h.

**Results:**

Twenty patients were randomized to senicapoc and 26 patients to standard care. Important differences existed in patient characteristics at baseline, including more patients being on non‐invasive/invasive ventilation in the control group (54% vs. 35%). The median senicapoc concentration at 72 h was 62.1 ng/ml (IQR 46.7–71.2). The primary outcome, PaO_2_/FiO_2_ ratio at 72 h, was significantly lower in the senicapoc group (mean 19.5 kPa, SD 6.6) than in the control group (mean 24.4 kPa, SD 9.2) (mean difference −5.1 kPa [95% CI −10.2, −0.04] *p* = .05). The 28‐day mortality in the senicapoc group was 2/20 (10%) compared with 6/26 (23%) in the control group (OR 0.36 95% CI 0.06–2.07, *p* = .26).

**Conclusions:**

Treatment with senicapoc resulted in a significantly lower PaO_2_/FiO_2_ ratio at 72 h with no differences for other outcomes.

**Editorial Comment**

In this phase 2 trial, for effect of senicapoc on the PaO2/FiO2 ratio in patients with COVID‐19 and severe respiratory insufficiency, the primary outcome at 72 h showed potential harm, however with no difference for other outcomes. The study was limited by the small sample size and imbalance in baseline characteristics.

## 1 INTRODUCTION

Coronavirus disease 2019 (COVID‐19) is caused by the severe acute respiratory syndrome coronavirus 2 (SARS‐CoV‐2).^1^, ^2^ COVID‐19 is primarily characterized by upper or lower respiratory tract symptoms.^3^ Although the majority of COVID‐19 cases are asymptomatic or only have mild disease, some patients require respiratory support such as high flow oxygen therapy or mechanical ventilation.^4^ In these patients, short‐term mortality rates are reported around 30%.^5^, ^6^

Interleukin‐6 receptor inhibitors and glucocorticoids, both targeting an excessive inflammatory response, have been reported to improve survival among severely ill patients in large randomized trials.^7^, ^8^, ^9^ However, no therapies have to date demonstrated an effect of directly targeting the pulmonary tissue injury induced by SARS‐CoV‐2. Preclinical studies suggest that ion channels situated in the lung's endothelial and epithelial cell layers play a crucial role in activating an inflammatory response and fluid transport across the alveolar‐capillary barrier.^10^, ^11^, ^12^ The calcium‐activated potassium channel of intermediate conductance (KCa3.1) is an ion channel highly expressed in the epithelium and white blood cells. It is a key regulator of fluid transport and inflammatory processes.^13^, ^14^, ^15^, ^16^ In a recent study in a mouse model of acute respiratory distress syndrome (ARDS), we found that a single dose of a KCa3.1 channel blocker, senicapoc, improved gas exchange measured as arterial‐to‐inspired oxygen (PaO_2_/FiO_2_) ratio, attenuated reduction in lung compliance, and diminished the pulmonary inflammatory response e.g., reduced neutrophil recruitment and pro‐inflammatory cytokine release.^17^ Furtermore, senicapoc protected against changes in the alveolar‐capillary barrier permeability and reduced neutrophil recruitment in a porcine models of ARDS.^17^, ^18^ Moreover, senicapoc has been reported to inhibit replication of the arenavirus.^19^ In phase III clinical trials for sickle cell anemia, senicapoc was found to be safe and well‐tolerated in non‐critically ill patients.^20^, ^21^ Therefore, blocking KCa3.1 activity with senicapoc could be a potential therapeutic strategy to treat respiratory failure in COVID‐19 patients in the ICU.

The objective of this trial was to determine if administration of enteral senicapoc improves the PaO_2_/FiO_2_ ratio after 72 h in intensive care unit (ICU) patients with COVID‐19 and respiratory insufficiency. We hypothesized that administration of enteral Senicapoc would improve the PaO_2_/FiO_2_ ratio at 72‐h in ICU patients with COVID‐19 and respiratory insufficiency.

## 2 METHODS

### 2.1 Study design

We conducted an investigator‐initiated, randomized, open‐label, phase II trial at four hospitals in Denmark. The full trial protocol is provided in the Supplemental Material. The trial was approved by the regional ethics committee (case number: 1‐10‐72‐84‐20) and the Danish Medicine Agency (EudraCT Number: 2020‐001420‐34). The study was conducted in accordance with Good Clinical Practice guidelines and the Declaration of Helsinki. According to Danish law, patients were enrolled based on an emergency basis (e.g., with consent from a doctor who was independent of the trial). Subsequently, written consent was obtained from a surrogate. Finally, written consent was obtained from the patient when able.

### 2.2 Participants

Patients were included if they were aged ≥18 years and admitted to an ICU with severe respiratory insufficiency due to COVID‐19. COVID‐19 was defined as a positive polymerase chain reaction (PCR) test for SARS‐CoV‐2, within 14 days prior to ICU admission. Severe respiratory insufficiency was defined as requiring supplemental oxygen ≥10 L/min or mechanical ventilation with an FiO_2_ ≥ 40%. Exclusion criteria were severe heart failure (ejection fraction <30%), severe renal insufficiency (eGFR <30 ml/min/1.73 m^2^), severe hemodynamic instability (noradrenalin dose >0.3 μg/kg/min), prior enrollment in the trial, pregnancy, allergy to senicapoc, inability to take enteral medication, more than 24 h since ICU admission, limitations of care, and anticipated death within 24 h. During the study period, on Sept. 23, 2020 (6 patients included at that time), the exclusion criteria “more than 24 h since ICU admission” was changed from “more than 12 h since ICU admission” due to prolonged response times for confirmation of coronavirus in PCR‐base tests.

### 2.3 Randomization

Eligible patients were randomized in a 1:1 ratio to either enteral senicapoc in addition to standard of care or standard of care alone in blocks with random sizes of 2 or 4. The randomization was stratified according to the baseline PaO_2_/FiO_2_ ratio (above or below 20 kPa [150 mmHg]) and site. The randomized allocation list was created by an independent statistician using a random number generator (SAS version 9.4 [SAS Institute, Cary, NC, USA]).

### 2.4 Intervention

The intervention consisted of 50 mg enteral senicapoc (5 × 10 mg tablets) administered as soon as possible after randomization and again after 24 h. Senicapoc was administered enterally as it is not available as an intravenous drug. Patients in the control group received standard care only. Physicians, patients, and individuals who assessed the outcomes were not blinded to the assigned treatment. All clinical interventions were left at the discretion of the clinical team for both groups. The level of oxygen therapy and the oxygen level being targeting was determined by the treating ICU physician independent of the trial. Senicapoc is not labeled for the treatment of COVID‐19 and the product is still investigational.

### 2.5 Clinical and laboratory data

Data on demographic characteristics were collected at inclusion, while laboratory values and physiological variables were collected daily for the first 10 days. The use of mechanical ventilation and other oxygen supportive therapies, including neuromuscular blocking agents, prone positioning, and extracorporeal membrane oxygenation (ECMO), were collected daily through day 10. The radiographic assessment of lung edema (RALE) score was used to evaluate baseline chest *X*‐rays.^22^

### 2.6 Outcomes

The primary outcome was the PaO_2_/FiO_2_ ratio 72 h after randomization. The ratio was calculated based on the PaO_2_ from the arterial gas and the concomitant FiO_2_. For patients on invasive or non‐invasive mechanical ventilation or supplemental oxygen with flow ≥15 L/min, the actual FiO_2_ value from the ventilator was used. In patients with flow <15 L/min, the FiO_2_ was estimated from conversion tables provided in the Supplemental Material.

Pre‐specified secondary clinical outcomes included ventilator‐free days within 28 days and 28‐day mortality. For definition of ventilator‐free days see Supplement Material.

Additional outcomes included vasopressor‐free days, need for renal replacement therapy within 28 days, and health‐related quality of life (EQ‐5D‐5L) at 28 days.^23^ For patients unable to respond to the EQ‐5D‐5L questionnaire due to health reasons (e.g., respiratory insufficiency, incompetent, still on mechanical ventilation) we assigned worst values. For EQ‐5D‐5L, an index value based on Danish data was calculated using the “Crosswalk Index Value Calculator.”^24^

Blood samples for measurement of senicapoc plasma concentrations and SARS‐CoV‐2 were drawn at baseline and after 24, 48, 72, 120, and 168 h. Blood samples were collected in EDTA tubes, centrifuged for 10 min at 3000 rpm, and the plasma was stored at −80 °C. A rapid, sensitive liquid chromatography–tandem mass spectrometry (LC–MS/MS) method was used to quantify senicapoc.^25^ SARS‐CoV2 was measured by PCR in plasma, see Supplement Material.^26^

### 2.7 Adverse events

To assess specific potential adverse events, we collected data on the following: cardiac arrhythmias, vasopressor refractory shock, allergic reaction, acute coronary syndrome, anemia, leucopenia, and hyperglycemia. For definitions, see Supplementary Material.

### 2.8 Sample size

The sample size was based on the primary outcome of the PaO_2_/FiO_2_ ratio at 72 h. Given the novelty of COVID‐19 at the time of protocol writing, there were limited data to support a definitive sample size calculation. Based on preliminary data,^3^, ^27^, ^28^ we anticipated a PaO_2_/FiO_2_ ratio of 120 mmHg (16 kPa) in the control group and 180 mmHg (24 kPa) in the senicapoc group. With a common standard deviation of 70 mmHg (9 kPa), an alpha of 5%, and based on a *t*‐test, 46 patients were needed to have 80% power to detect a statistically significant difference.

### 2.9 Statistical analysis

Continuous variables are presented as medians with interquartile range (IQR) and categorical variables as counts with frequencies. The primary outcome, the PaO_2_/FiO_2_ ratio at 72 h, was compared between groups using linear regression adjusting for the two stratification variables (baseline PaO_2_/FiO_2_ ratio and site) as fixed effects. Results are presented as mean differences with 95% confidence intervals. As a post hoc subgroup analysis, the PaO_2_/FiO_2_ ratio at 72 h was compared separately in patients on supplemental oxygen and in patients on non‐invasive/invasive ventilation at baseline. Binary outcomes (mortality) were compared between groups with logistic regression adjusting only for baseline PaO_2_/FiO_2_ due to the low number of events. The other continuous variables were compared using the Wilcoxon rank‐sum test (unadjusted analysis) and the van Elteren test (a stratified extension of the Wilcoxon Rank Sum test) as the data were substantially skewed.^29^ Categorical variables were compared using Fisher's exact test. With regards to EQ‐5D‐5L, we performed two post hoc explanatory sensitivity analyses (1) dead patients were assigned the worst value and included in the analysis and (2) including only patients able to answer the EQ‐5D‐5L questionnaire. A change in number of viral copies of SARS‐CoV2 was analyzed using a repeated measurements mixed‐effects model. Because of the potential for Type I error due to multiple comparisons, the analyses of the secondary endpoints should be considered exploratory. All tests were two‐sided, a *p*‐value <.05 was considered significant, and all confidence intervals have 95% coverage. Stata software, version 16 (StataCorp, College Station, TX, USA) was used for the analyses.

## 3 RESULTS

### 3.1 Trial flow and baseline characteristics of participants

**FIGURE 1:**
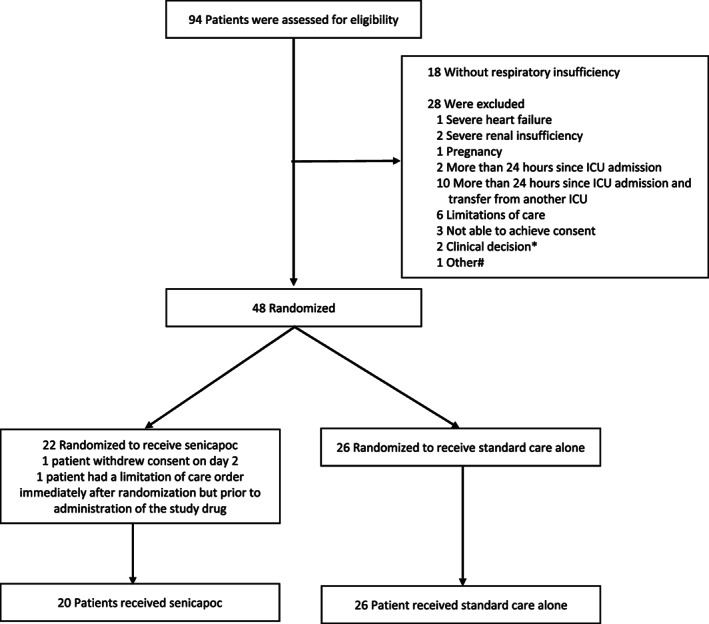
Participant Flow. Randomization was stratified by site and baseline PaO_2_/FiO_2_ ratio (above or below mmHg 20 kPa). No patient was lost to follow‐up. *Renal transplant patients, renal consultant worried about potential interactions with immunosuppressant. ^#^Admitted for other diseases than COVID‐19

From 28th of April 2020 to 28th of December 2020, 48 patients were randomized. Two patients were excluded from the trial. One patient withdrew consent and one patient received a limitation of care order immediately after randomization and before trial drug administration (Figure 1). Of the included patients, 20 were randomized to senicapoc, while 26 were randomized to the control group. The trial groups had similar characteristics regarding age, body mass index, the interval between ICU admission and randomization, and the majority of coexisting illnesses (Table 1). More patients in the senicapoc group had diabetes, while more patients in the control group were men and had a higher Sequential Organ Failure Assessment (SOFA) Score and RALE score. Patients in the two groups were balanced with regards to COVID‐19 disease characteristics, including symptoms, COVID‐19 treatments, and enrollment into other trials (eTable 1). More patients in the control group were on non‐invasive/invasive ventilation at baseline (54% vs. 35%); otherwise, groups were balanced at baseline for respiratory and arterial blood gas parameters (eTable 2).

**TABLE 1:** Characteristics of the patients at baseline

Variables	COVIPOC group (*n* = 20)	Control group (*n* = 26)
Age median (IQR)—yr	66 (58–70)	66 (56–74)
Male sex—no. (%)	10 (50)	20 (77)
Body mass index	27.7 (25–31)	31.3 (26.4–33.2)
The median interval between ICU admission and randomization (IQR)—hours	11.3 (8.7–16.7)	15.1 (9.8–19.2)
Coexisting cardiovascular illness mmHg—no. (%)		
Coronary artery disease	1 (5)	4 (15.4)
Chronic heart failure	1 (5)	1 (3.9)
Atrial fibrillation	2 (10)	4 (15.4)
Stroke	0 (0)	0 (0)
Venous thromboembolism	0 (0)	1 (3.9)
Hypertension	7 (35)	9 (34.6)
Coexisting non‐cardiovascular illness—no. (%)		
Diabetes	8 (40)	5 (19.2)
Pulmonary disease	5 (25)	7 (26.9)
Renal disease	0 (0)	2 (7.7)
Liver disease	0 (0)	0 (0)
Cancer	2 (10)	5 (19.2)
Dementia	0 (0)	0 (0)
Median SOFA score (IQR)	3 (2–5)	6.5 (2–10)
Median Frailty score prior to acute illness (IQR)	3 (2–3)	3 (3–4)
Median RALE score (IQR)^a^	10 (4–21)	14 (9–24)
PaO_2_/FiO_2_ ratio (kPa)—mean (SD)	14.2 (7.3)	15.5 (5.6)
Supplemental oxygen only—no. (%)	13 (65)	12 (46)
Non‐Invasive/invasive ventilation—no. (%)	7 (35)	14 (54)

Scores on the Sequential Organ Failure Assessment (SOFA) range from 0 to 24 with higher scores indicating more severe organ failure. ICU: Intensive care unit. IQR: Interquartile range. SD: Standard deviation: RALE: The radiographic assessment of lung edema score.

a Three patients did not have a chest x‐ray before randomization, why 2 patients in the control group and 1 patient in the senicapoc group have a missing RALE score.

### 3.2 Senicapoc

**FIGURE 2:**
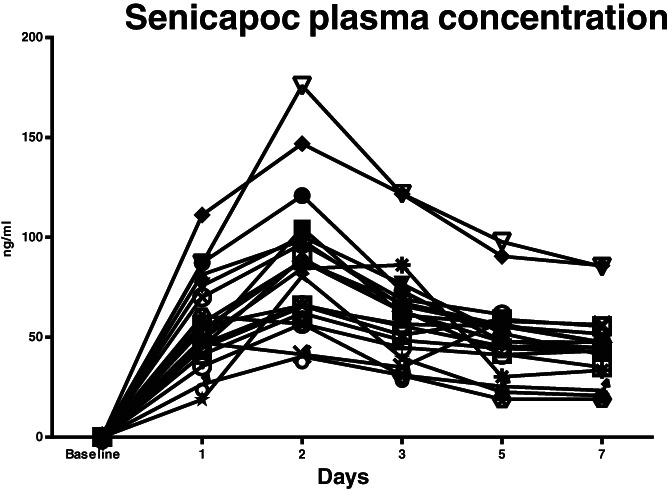
Senicapoc plasma concentrations from baseline to day 7 in the intervention group (*n* = 20). One patient had missing samples at day 7

In controls, the senicapoc concentration was below the detection limit in all samples. Senicapoc concentrations in the intervention group is displayed in Figure 2. The median senicapoc concentration at 24 h was 51.4 ng/ml (IQR 44.9–72.9), 86.3 ng/ml (IQR 63.7–99.5) at 48 h, and 62.1 ng/ml (IQR 46.7–71.2) at 72 h. One patient had missing blood samples at day 7.

### 3.3 Primary outcome

**FIGURE 3:**
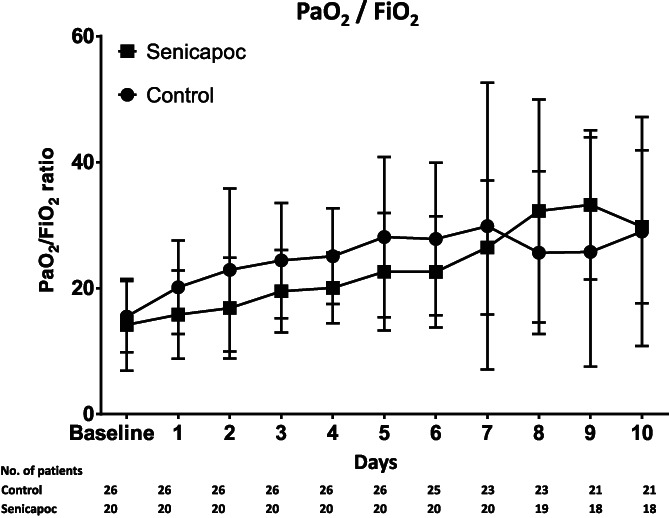
Mean PaO_2_/FiO_2_ ratio from baseline to day 10 in the two groups. All patients with a missing PaO_2_/FiO_2_ ratio from day 8 and forward were discharged home, except for one patient in the standard care group dying at day 8. Error bars SD

The PaO_2_/FiO_2_ ratio was significantly lower in the senicapoc group at 72 h than in the control group (Table 2). A post hoc explanatory analysis showed that this difference was restricted to patients on supplemental oxygen therapy at baseline (mean difference 9.6 [95% CI 1.37–17.9] *p* = .03), with no difference in patients on non‐invasive/invasive ventilation at baseline (mean difference −1.65 [95% CI −8.4 to 5.1] *p* = .6). When looking at the PaO_2_/FiO_2_ ratio over time, it was lower in the senicapoc group from baseline to day 8 but higher from day 8 and forward (Figure 3). All patients with a missing PaO_2_/FiO_2_ ratio from day 8 and forward were discharged home, except for one patient in the standard care group dying at day 8 (Figure 3).

**TABLE 2:** Outcomes

Primary outcome	Senicapoc group (*n* = 20)	Control group (*n* = 26)	Unadjusted analysis	Adjusted analysis
			Senicapoc group (*n* = 20)	Control group (*n* = 26)
PaO_2_/FiO_2_ ratio (kPa) 72 h—Mean (SD)	19.5 (6.6)	24.4 (9.6)	Mean difference −4.9 (95% CI −9.8 to −0.0) *p* = .049	Mean difference −5.1 (95% CI −10.2 to −0.0) *p* = .048^a^
*Secondary outcomes*				
Ventilator‐free hours—Median (IQR) hours	607 (398 to 672)	486 (0 to 672)	*p* = .19^b^	*p* = .15^c^
28‐day mortality—no. (%)	2 (10)	6 (23)	OR 0.37 (95% CI 0.07 to 2.07) *p* = .26	OR 0.36 (95% CI 0.06 to 2.07) *p* = .26^d^
*Other outcomes*				
Vasopressor‐free hours—Median (IQR)	672 (627 to 672)	616 (490 to 672)	*p* = .07^b^	*p* = .08^c^
Renal replacement therapy—no. (%)	0 (0)	5 (19.2)	*p* = .06^e^	
SOFA score 72 h—Median (IQR)	4.5 (2 to 7.5)	7 (2 to 10)	*p* = .35^b^	*p* = .32^c^
Median health‐related quality of life (EQ‐5D‐5L) index score (IQR)^f^	53 (47 to 69)	69 (−29 to 79)	*p* = .48^b^	*p* = 1.0^c^
Median EQ VAS (IQR)^f^	50 (40 to 65)	45 (7.5 to 72.5)	*p* = .77^b^	*p* = .76^c^

Abbreviations: IQR, interquartile range; SD, standard deviation; SOFA: sequential organ failure assessment.

^a^ Adjusted for site and baseline PaO_2_/FiO_2_ ratio.

^b^ Wilcoxon rank sum test.

^c^ Van Elteren's test stratified by baseline PaO_2_/FiO_2_ ratio <20 kPa or ≥20 kPa.

^d^ Due to low number of outcomes only adjusted for baseline PaO_2_/FiO_2_ ratio.

^e^ Fisher's exact test.

^f^ Including 18 patients in the senicapoc group and 20 in the control group. 1 patient in senicapoc group and 5 patients in the control group had worst values assigned.

### 3.4 Secondary outcomes

Secondary outcomes are listed in Table 2. The number of ventilator‐free days was not different between groups. Two out of twenty patients (10%) in the senicapoc group died before day 28, while 6 out of 26 (23%) patients died in the control group. Five patients in the control group required renal replacement therapy, with no patients in the senicapoc group requiring renal replacement therapy during the study. Health related quality of life evaluated by EQ‐5D‐5L was comparable between groups (Table 2/eTable 3).

Laboratory values from baseline to day 10 are presented in the supplement (eFigure 1–8). Groups were comparable except for lower levels of leukocytes, neutrophils and creatinine in the senicapoc group throughout the study period.

### 3.5 Respiratory parameters and arterial blood gas values

Respiratory parameters and arterial blood gas values are shown in eTable 2. The higher number of patients on non‐invasive/invasive ventilation in the control group at baseline was constant for the first 72 h. Ventilator settings and arterial blood gas values were in general comparable between the two groups.

### 3.6 SARS‐CoV2 was measured by PCR

The number of SARS‐CoV2 viral copies are displayed in eFigure 9. There was no change in number of copies over time or between groups.

### 3.7 Adverse events

Specific adverse events are shown in eTable 4. Specific adverse events were low in both groups with no serious adverse events attributed to the study treatment.

## 4 DISCUSSION

In this trial, senicapoc treatment in patients admitted to the ICU due to severe respiratory insufficiency resulted in a statistically significantly lower PaO_2_/FiO_2_ ratio. There were no differences in the secondary outcomes.

The current clinical trial is the first investigating the effect of senicapoc administered to patients with severe respiratory insufficiency. Previous clinical trials of senicapoc have only included outpatients with sickle cell disease or asthma with a limited number of treatment‐related adverse effects.^21^, ^30^ In contrast, patients with severe respiratory insufficiency admitted to the ICU are at high risk of organ failure, and there is increased concern regarding the interaction with other drugs. In general, the reported number of specific adverse events was low, with no serious adverse events attributed to the study drug. These observations should, of course, be interpreted in light of the small sample size. The safety of the intervention is therefore still unknown.

Senicapoc was administered orally or administered through a gastric feeding tube. Based on previous dose‐findings studies, a plasma concentration of 56.5 ng/ml and 115.5 ng/ml was anticipated at 24 and 48 h. Although slightly lower plasma concentrations were measured, the achieved concentrations were above the desired concentration of 3.23 ng/ml.^31^ The study was therefore successful in obtaining the desired plasma concentrations.

Contrary to our hypothesis of an improvement in PaO_2_/FiO_2_ ratio, we observed a significantly lower PaO_2_/FiO_2_ ratio in the senicapoc group at 72 h. Although the study was randomized and stratified according to site and baseline PaO_2_/FiO_2_ ratio, more patients in the control group were on non‐invasive/invasive ventilation at baseline. These findings indicate increased disease severity in the control group, also illustrated by a higher SOFA score and a higher RALE score at baseline. In light of a significantly higher PaO_2_/FiO_2_ ratio in the control group, this may be seen as an additional argument against a protective effect of senicapoc. However, in open systems, the FiO_2_ value may be significantly overestimated when using standardized conversion tables, resulting in a falsely low PaO_2_/FiO_2_ ratio.^32^, ^33^ As a higher percentage of patients in the senicapoc group were on open systems, this may have resulted in a lower PaO_2_/FiO_2_ ratio in the senicapoc group. Although this approach of using standardized conversion tables has been employed by other studies^33^ they included a different patient population and whether this conversion also applies to patients with COVID‐19 is unknown.

The hypothesis of a protective effect of senicapoc in patients with severe respiratory insufficiency was based on a strong physiological rationale. Animal studies demonstrated improved gas exchange, attenuated reduction in lung compliance, and an attenuated pulmonary inflammatory response (e.g., reduced neutrophil recruitment and pro‐inflammatory cytokine release) even after a single dose senicapoc.^18^, ^34^ These findings, along with studies demonstrating an augmented inflammatory response and a reduction in lung compliance in patients with severe COVID‐19, supports that senicapoc could have had a potential protective effect.^35^, ^36^, ^37^ However, two important differences exist. Several studies have compared COVID‐19 induced ARDS with classical ARDS,^37^, ^38^ and there is increasing evidence suggesting that pulmonary thrombosis is an important part of the pathophysiology of COVID‐19.^39^, ^40^, ^41^ This aspect of pulmonary thrombosis is not included in animal models of classic ARDS induced by high‐volume mechanical ventilation combined with saline lavage. Furthermore, with the publication of studies demonstrating a protective effect of corticosteroids, dexamethasone was implemented as a standard of care.^8^, ^42^ Consequently, more than 90% of the included patients in both groups received dexamethasone, which could have dampened a potential protective anti‐inflammatory effect of senicapoc, as dexamethasone was not included as a standard of care therapy in the original animal studies.^18^, ^34^

Although the primary endpoint favored standard care, several secondary endpoints pointed towards a protective effect of senicapoc, including 28‐day mortality and the number of patients receiving renal replacement therapy. However, none of the differences were statistically significant, and in any case, the comparisons should be considered explorative, only. The higher ventilator‐free hours and vasopressor‐free hours in the senicapoc group should be interpreted in the light of a lower number of patients being on non‐invasive/invasive ventilation at baseline. A higher number of patients in the senicapoc group were therefore never exposed to non‐invasive/invasive ventilation and as consequence of this vasopressor therapy as vasopressor therapy is more frequently used in sedated mechanically ventilated patients.

The current trial has important strengths. The trial was completed within a short period of time, in a setting without a significant burden on the health care system and a constant mortality rate during the entire pandemic.^5^, ^43^ There was no loss to follow‐up, and detailed data on respiratory parameters and laboratory data were included.

The trial also has some limitations. The study was open‐label which increases the risk of bias. The PaO_2_/FiO_2_ ratio was chosen as it is a commonly used measure of illness severity in patients with ARDS and is used to define the degree of severity.^44^ Furthermore, the PaO_2_/FiO_2_ ratio is associated with mortality,^44^, ^45^ making it a potentially useful surrogate outcome for phase II trials.^46^ However the use of surrogate outcomes may lead to larger treatment effects compared to patient relevant outcomes.^47^ The inclusion of a post hoc subgroup analysis is purely explanatory and should be viewed in that light. Furthermore, as patients with COVID can have a protracted course of the disease, evaluating the effect of senicapoc at 72 h might have been premature. Although patients were randomized in a 1:1 ratio in blocks with random sizes of 2 or 4 and stratified by site, more patients were randomized to standard care than the control group. Also, considerable imbalances existed at baseline which is a limitation. This is likely caused by the small study size, one site recruiting a limited number of patients, and 2 patients being excluded in the senicapoc group after randomization. Exclusion of patients after randomization may have introduced bias.

## 5 CONCLUSION

Treatment with senicapoc resulted in a significantly lower PaO_2_/FiO_2_ ratio at 72 h with no difference for other outcomes.

### Trial registration

EU Clinical Trials Register nr. 2020‐001420‐34. Date of registration 2020‐04‐01. https://www.clinicaltrialsregister.eu/ctr-search/trial/2020-001420-34/DK

**Abbreviations**
ARDS: acute respiratory distress syndrome
PaO_2_/FiO_2_
: arterial‐to‐inspired oxygen
COVID‐19: Coronavirus disease 2019
ECMO: Extracorporeal membrane oxygenation
EQ‐5D‐5L: health‐related quality of life
ICU: intensive care unit
PCR: polymerase chain reaction
RALE: radiographic assessment of lung edema
SARS‐CoV‐2: severe acute respiratory syndrome coronavirus 2

## AUTHOR CONTRIBUTIONS

AG, LWA, MFV, and US wrote the protocol with input form the steering comitee (OH, SC, KJK, IJ, TS, BSD OS). AG, AGM, SC, BSD, OS, TS, IJ, and KJK were involved in patient inclusion and data collection. SM JBH and LBH analyzed patient blood samples. AG, LWA, MFV, and US were involved in the analysis of the data. All authors contributed to critical reading of the text and its revision. All authors read and approved the final manuscript.

## Supporting information


Appendix S1

## Data Availability

Six months after the publication of the results, all deidentified individual patient data will be made available for data sharing.
