# Supplementary material for: Senicapoc treatment in COVID‐19 patients with severe respiratory insufficiency—A randomized, open‐label, phase II trial
Source: Acta Anaesthesiol Scand. 2022 May 13;66(7):838–46. doi: 10.1111/aas.14072 (PMC9111301; doi:10.1111/aas.14072)
Supplement: Supplementary file 1 — Appendix S1 [file AAS-66-838-s001.pdf]

Senicapoc in COVID-19 Patients with Severe Respiratory Insufficiency

– A Randomized, Open-Label, Phase II Trial

Supplement

Contents

Ventilator-free days 2
eTable 1 COVID-19 specific characteristics 3
eTable2 Respiratory and arterial blood gas parameters 5
eTable 3 Post hoc explanatory analysis 6
eTable 4. Number of patients with a specific adverse events — no. (%) 7
eFIgure 1 C-reactive protein 8
eFigure 2 - Leukocytes 9
eFigure 3 - Neutrophils 10
eFigure 4 - Lymphocytes 11
eFigure 5 - Hemoglobin 12
eFIgure 6 – Creatinine 13
eFigure 7 - Platelets 14
eFigure 8 - Glukose 15
eFigure 9 Virus Load 16
Detection of SARS-CoV2 in plasma 17
Fraction of inspired oxygen (FiO2) conversion tables 18
Protocol 19

Ventilator-free days

Ventilator-free days was defined as the number of days within the first 28 days after randomization where the patient was alive and not on invasive mechanical ventilation. If the patient died within 28 days, the number of ventilator-free days was set to zero.

eTable 1 COVID-19 specific characteristics		
	**COVIPOC group (n=20)**	**Control group (n=26)**
Median days from symptom onset to hospital admission (IQR)[a]	8 (5-9)	8 (4-10)
Median days from positive SARS-CoV-2 to ICU admission (IQR)	10 (9-12)	10 (4-13)
Symptoms at admission — no. (%)		
Cough	12 (60)	11 (42.3)
Sore throat / throat pain	3 (15)	3 (11.5)
Dyspnea	18 (90)	17 (65.4)
Muscle pain	5 (25)	8 (30.8)
General feeling of being unwell	15 (75)	16 (61.5)
Running nose / rhinitis	0 (0)	1 (4)
Chills and/or night sweats	2 (10)	4 (15.4)
Gastrointestinal symptoms	4 (20)	4 (15.4)
Other symptoms	11 (55)	13 (50)
COVID-19-illness primary reason for admission — no. (%)[b]	20 (100)	25 (96)
COVID-19 specific therapies up to randomization — no. (%)		
Antibiotics	8 (40)	11 (42.3)
Dexamethasone	13 (65)	19 (73.1)
Remdesivir	8 (40)	12 (46.2)
Convalescent Plasma	0 (0)	1 (3.9)
Inclusion into other trials — no. (%)		
COVISTEROID / COVISTEROID 2[c]	8 (40)	12 (46.2)
CamoCO-19[d]	4 (20)	1 (3.9)
HOT-COVID[e]	6 (40)	8 (30.1)
CCAP2 trial[f]	0 (0)	1 (3.9)
Dexamethasone during ICU admission	18 (90)	24 (92.3)

a Excluding one patient infected during admission, as he had negative time from admission symptom onset

b One patient was infected with COVID-19 during admission for treatment of leukemia.

c Low dose hydrocortisone or 6 mg vs 12 mg dexamethasone in patients with COVID-19 and severe hypoxia, ClinicalTrials.gov Identifier NCT04509973
d Randomized trial of Camostat Mesilate a potent serine protease inhibitor or placebo, ClinicalTrials.gov Identifier NCT04321096
e PaO_2 of 8 kPa vs PaO_2 of 12 kPa in COVID-19 Patients With Acute Hypoxaemic Respiratory Failure in the Intensive Care Unit , ClinicalTrials.gov Identifier NCT04425031
f Convalescent anti-SARS-CoV-2 plasma trial, ClinicalTrials.gov Identifier NCT04345289
COVID-19: Coronavirus disease 2019. SARS-CoV-2: Acute respiratory syndrome coronavirus 2. ICU: Intensive care unit. IQR: Interquartile range

eTable2 Respiratory and arterial blood gas parameters								
	**Baseline**		**24 hours**		**48 hours**		**72 hours**	
	**Senicapoc group (n=20)**	**Control group (n=26)**	**Senicapoc group (n=20)**	**Control group (n=26)**	**Senicapoc group (n=20)**	**Control group (n=26)**	**Senicapoc group (n=20)**	**Control group (n=26)**
**Supplemental oxygen only**								
no. (%)	13 (65)	12 (46)	12 (60)	10 (39)	12 (60)	11 (4)	11 (55)	9 (35)
FiO_2 Median (IQR)	0.95 (0.75-1.0)	1 (0.61-1.0)	0.88 (0.66-1.0)	0.68 (0.62-1.0)	0.98 (0.49-1.0)	0.7 (0.4-0.9)	0.54 (0.44-1.0)	0.4 (0.35-0.7)
Flow Median (IQR) - l/min	20 (15-30)	13 (10-22)	25.5 (15-45)	15 (10-50)	15 (7-32)	29 (4-50)	10 (5-35)	4 (4-35)
**Non-Invasive / invasive ventilation**								
no. (%)	7 (35)	14 (54)	8 (40)	16 (62)	8 (40)	15 (58)	9 (45)	17 (65.4)
FiO_2 Median IQR	0.50 (0.45-0.8)	0.63 (0.5-0.75)	0.50 (0.43-0.71)	0.48 (0.4-0.6)	0.58 (0.38-0.82)	0.5 (0.4-0.55)	0.4 (0.35-0.7)	0.45 (0.35-0.5)
Tidal volume ml Median IQR	530 (359-570)	498 (440-520)	438 (355-589)	490 (408-570)	428 (357-481)	499 (450-613)	450 (377-466)	539 (450-655)
Respiratory frequency min^{-1} Median IQR	22 (21-27)	21 (18-24)	22.5 (21.5-25.5)	20 (16-20.5)	24.5 (22-26.5)	20 (18-23)	20 (20-22)	20 (17-21)
Peep cmH_20 Median IQR	10 (6-15)	12 (11-14)	11 (10-14)	12.5 (12-13.5)	12.5 (12-14)	12 (10-13)	10 (8-12)	12 (10-12)
Peak Pressure Median IQR	20 (10-32)	26 (22-28)	25 (14.5-30.5)	25.5 (21.5-27.5)	26.5 (22.5-30.0)	28 (25-31)	23 (21-28)	26 (23-29)
Position supine / prone no. (%)	1 (16.7)	2(14.3)	2 (25)	6 (37.5)	6 (75)	4 (26.7)	3 (33.3)	1 (5.9)
**Arterial blood gas values**								
pH Median IQR	7.44 (7.42-7.46)	7.43 (7.36-7.47)	7.44 (7.43-7.47)	7.42 (7.38-7.45)[a]	7.45 (7.41-7.47)	7.43 (7.4-7.48)[b]	7.46 (7.43-7.47)[c]	7.46 (7.43-7.49)[d]
$PaCO_2$ Median IQR	4.4 (4.2-5.4)	4.9 (4.1-6.0)	4.6 (4.3-5.6)	5 (4.6-5.7)	5 (4.5-5.4)	5.4 (4.6-6.1)	5 (4.5-5.7)	5.3 (4.7-5.8)
PaO_2 Median IQR	9.3 (8.8-10.2)	10.3 (8.4-12.3)	9.4 (8.8-10.5)	9.8 (8.9-11.5)	9.2 (8.8-11.2)	9.1 (8.7-11.2)	10.2 (9.1-11.6)	9.1 (8.7-10.9)
BE Median IQR	-0.1 (-2.2-1.7)	-0.1 (-2.5-0.9)	1.1 (-1.7-2.8)	0.8 (-2.1-1.8)	1.8 (-1.1-4.3)	1.4 (-1.3-3.6)	1.8 (0.8-4.9)	2.3 (0.7-4.8)
Lactate Median IQR	1.4 (1-1.7)	1.4 (1-1.8)	1.8 (1.4-2.3)	1.7 (1.2-2.5)	1.7 (1.2-2.8)	1.8 (1.1-2.5)	1.5 (1.3-2.9)	1.8 (1.3-2.4)
Respiratory SOFA score Median (IQR)	2 (2-2)	2 (2-3)	2 (2-3)	2 (2-3)	2 (2-3)	2 (2-3)	2 (2-3)	2 (2-3)

a One patient in the control group did not have an arterial gas taken at 24 hours
b Three patients in the control group did not have an arterial gas taken at 48 hours
c One patient in the senicapoc group did not have an arterial gas taken at 72 hours
d Two patients in the control group did not have an arterial gas taken at 72 hours

eTable 3 Post hoc explanatory analysis			**Unadjusted analysis**	**Adjusted analysis**
	**Senicapoc group (n=20)**	**Control group (n=26)**	**Senicapoc group (n=20)**	**Control group (n=26)**
Median Health-related quality of life (EQ-5D-5L) index score (IQR)[a]	50 (13-69)	13 (-62-75)	p=0.5[b]	p=41[c]
Median EQ VAS (IQR)[a]	50 (33-63)	19 (0-70)	p=0.3[b]	p=0.29[c]
Median Health-related quality of life (EQ-5D-5L) index score (IQR)[d]	55 (48-69)	73 (32-82)	p=0.11[b]	p=19[c]
Median EQ VAS (IQR)[d]	50 (40-65)	70 (35-85)	p=0.34[b]	p=0.36[c]

a Post hoc explanatory analysis where patients that had died had worst values assigned
b b Wilcoxon rank sum test
c Van Elteren´s test stratified by baseline PaO2/FiO2 ratio < 20 kPa or ≥20 kPa
d Post hoc explanatory analysis including only patients able to answer the EQ-5D-5L questionnaire. Senicapoc (n=17) and control (15)

eTable 4. Number of patients with a specific adverse events — no. (%)		
	**Senicapoc group (n=20)**	**Control group (n=26)**
Cardiac arrhythmia	2 (10)	6 (23)
Vasopressor-refractory shock	1 (5)	4 (15)
Allergic reaction, generalized	0 (0)	0 (0)
Myocardial infarction	0 (0)	0 (0)
Anemia	1 (5)	3 (12)
Leucopenia	1 (5)	2 (8)
Severe hyperglycemia	5 (25)	7 (27)

eFIgure 1 C-reactive protein

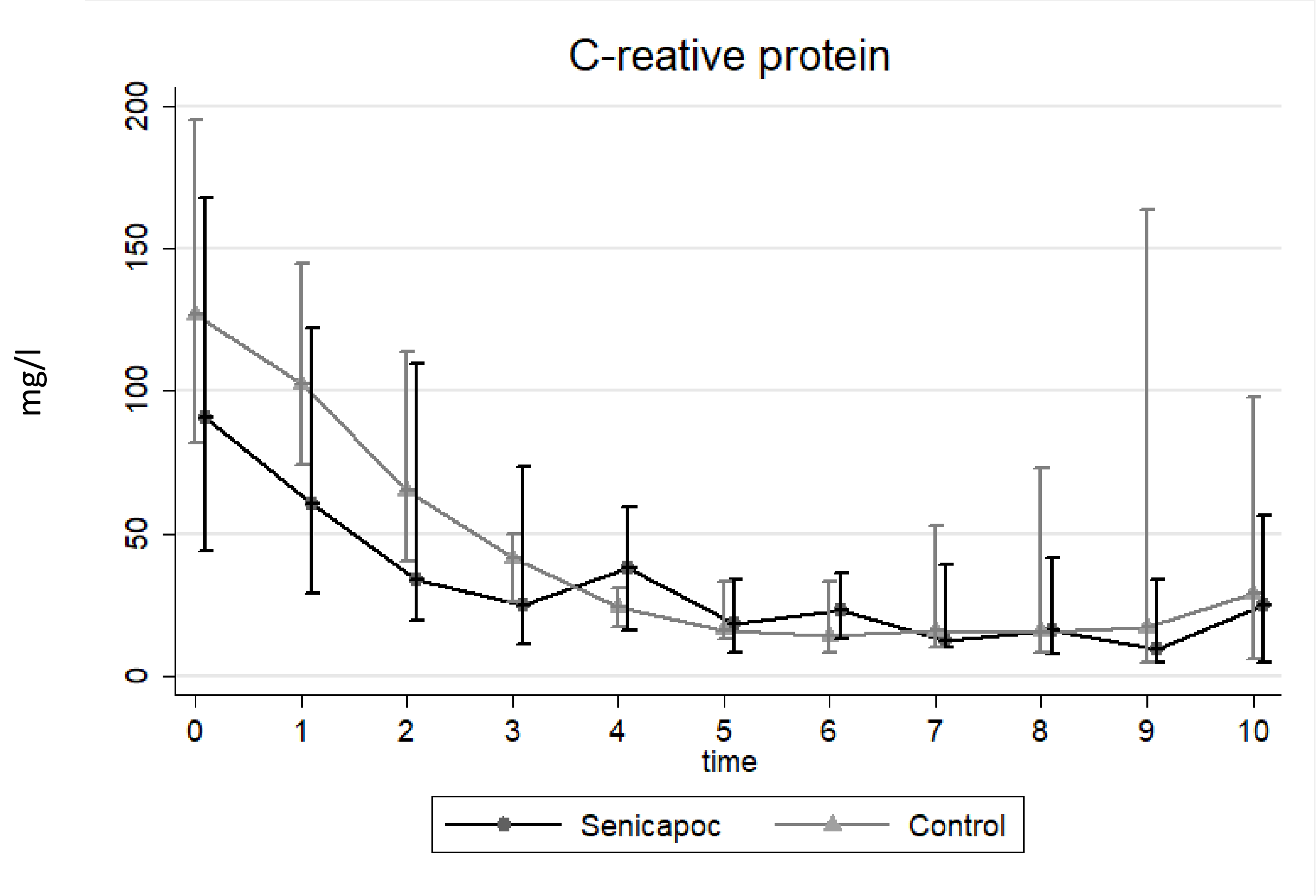

**No. of patients**											
**Control**	26	26	26	25	24	26	23	22	22	19	18
**Senicapoc**	20	20	20	20	18	20	15	17	16	16	14

Displayed as median (IQR)

eFigure 2 - Leukocytes

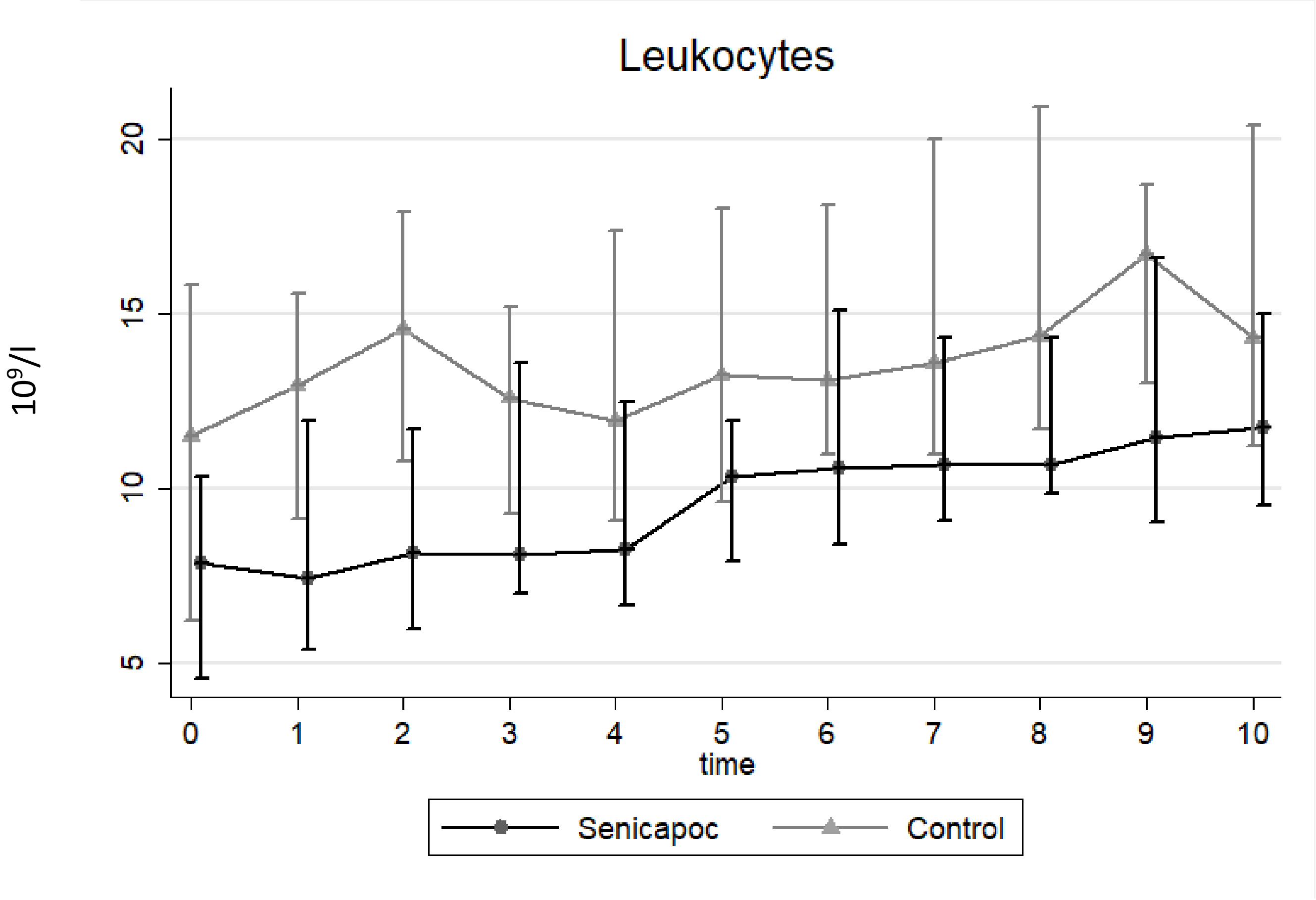

No. of patients											
**Control**	26	26	26	25	24	26	23	23	22	19	18
**Senicapoc**	20	20	20	20	18	20	15	17	16	16	13

Displayed as median (IQR)

eFigure 3 - Neutrophils

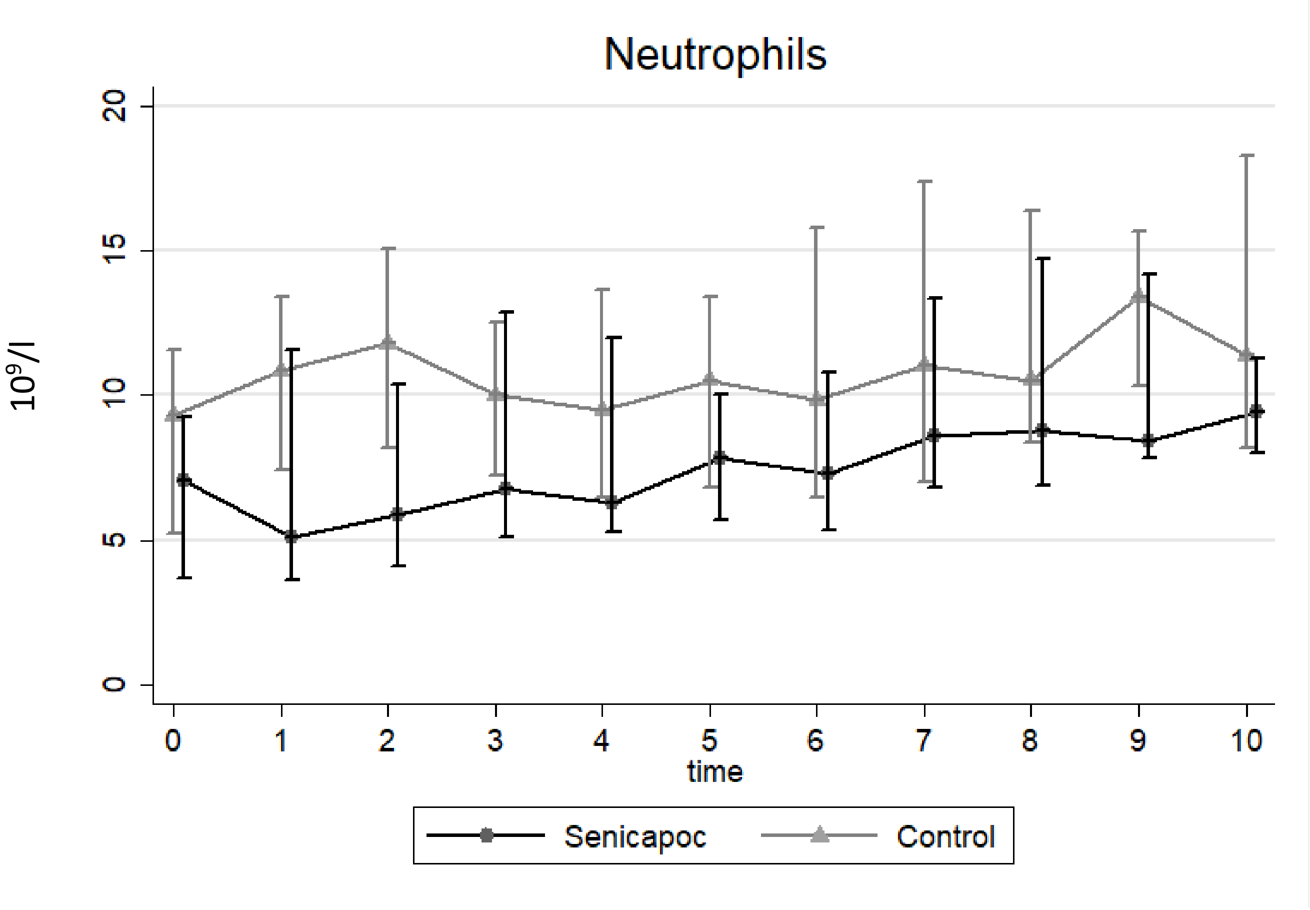

No. of patients											
**Control**	24	26	26	25	24	26	21	22	18	17	17
**Senicapoc**	19	19	19	19	17	18	12	15	15	15	12

Displayed as median (IQR)

eFigure 4 - Lymphocytes

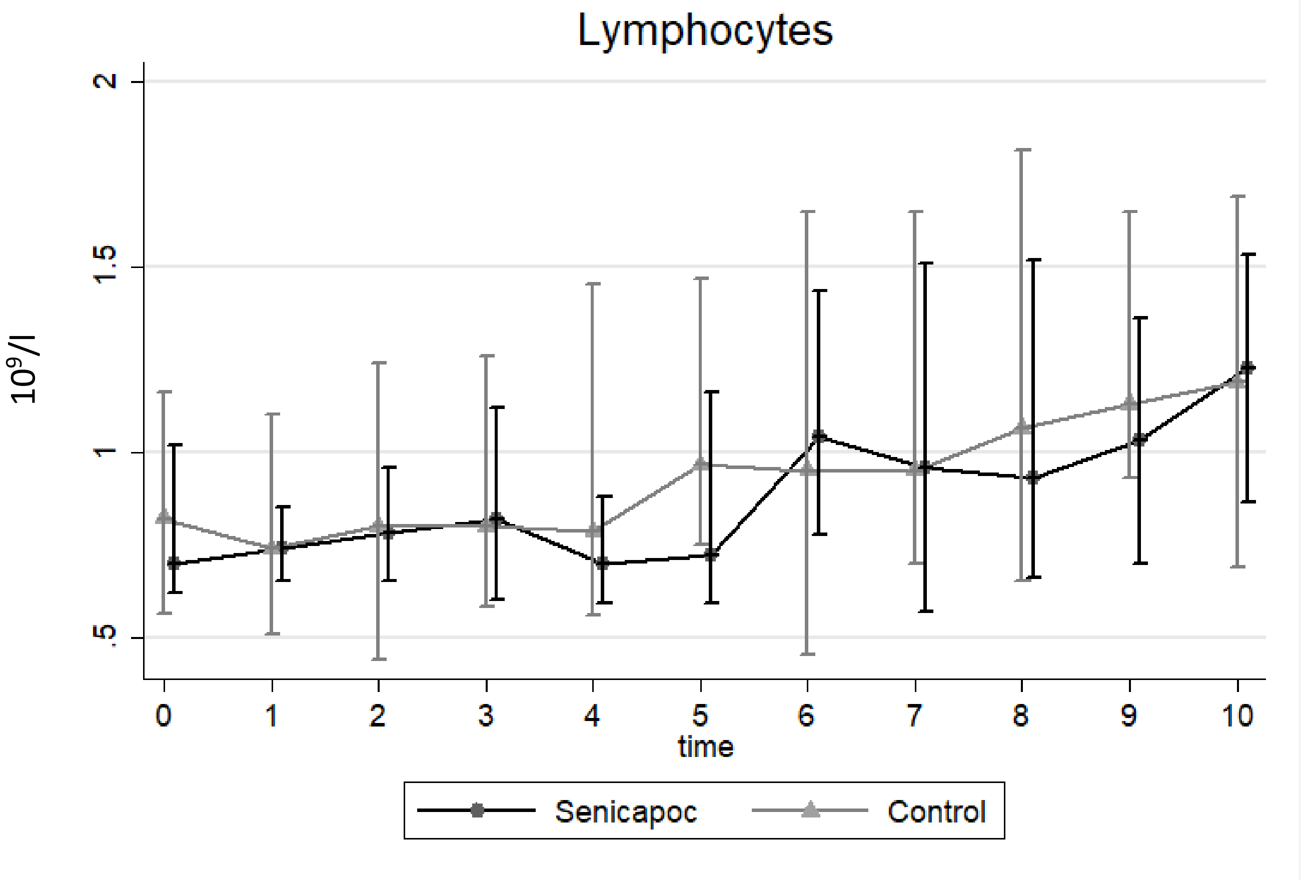

No. of patients											
**Control**	24	26	26	25	24	26	21	22	18	17	17
**Senicapoc**	19	19	19	19	17	18	12	15	15	15	12

Displayed as median (IQR)

eFigure 5 - Hemoglobin

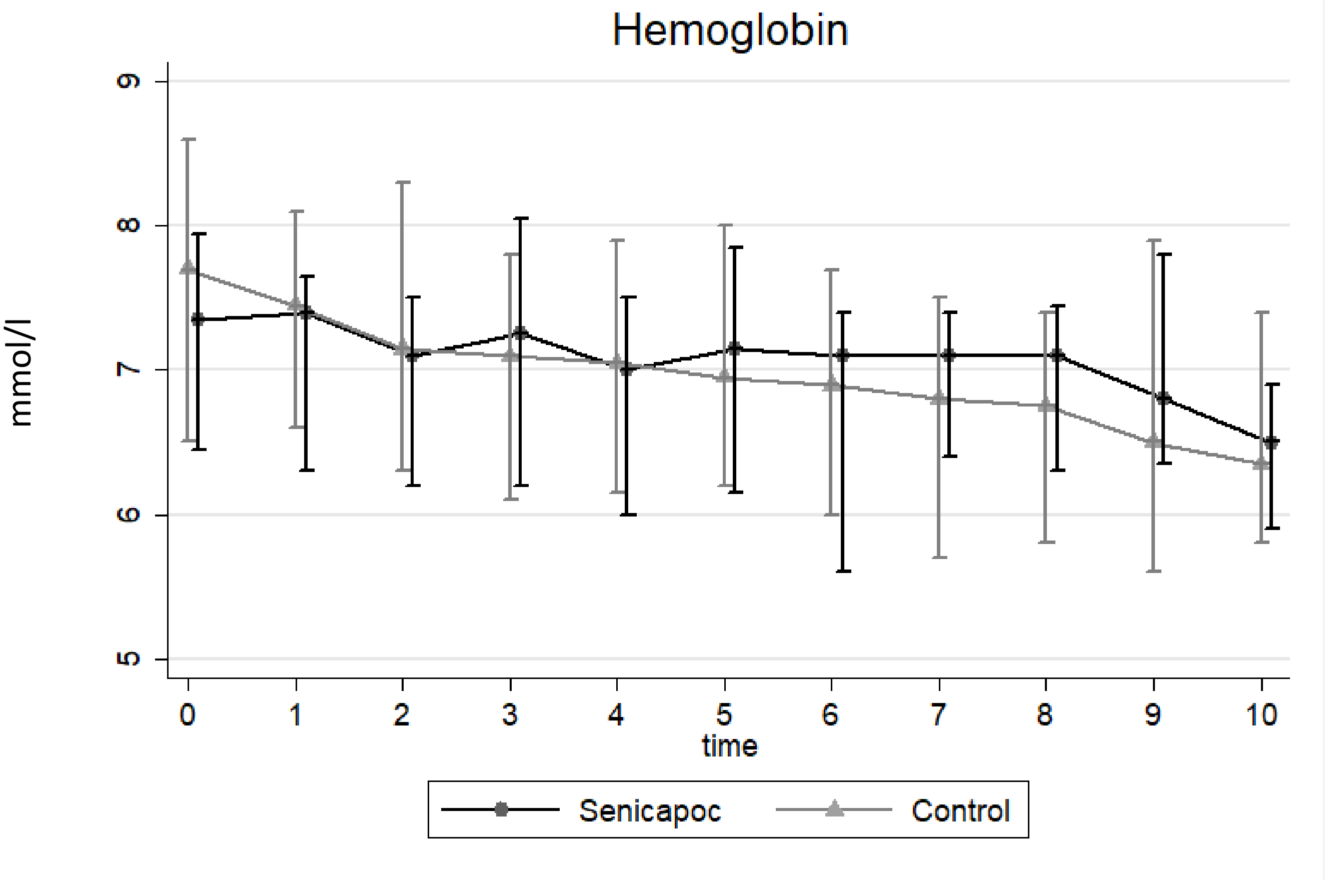

No. of patients											
**Control**	26	26	26	25	24	26	23	22	22	19	18
**Senicapoc**	20	20	20	20	18	20	15	17	16	16	14

Displayed as median (IQR)

eFIgure 6 – Creatinine

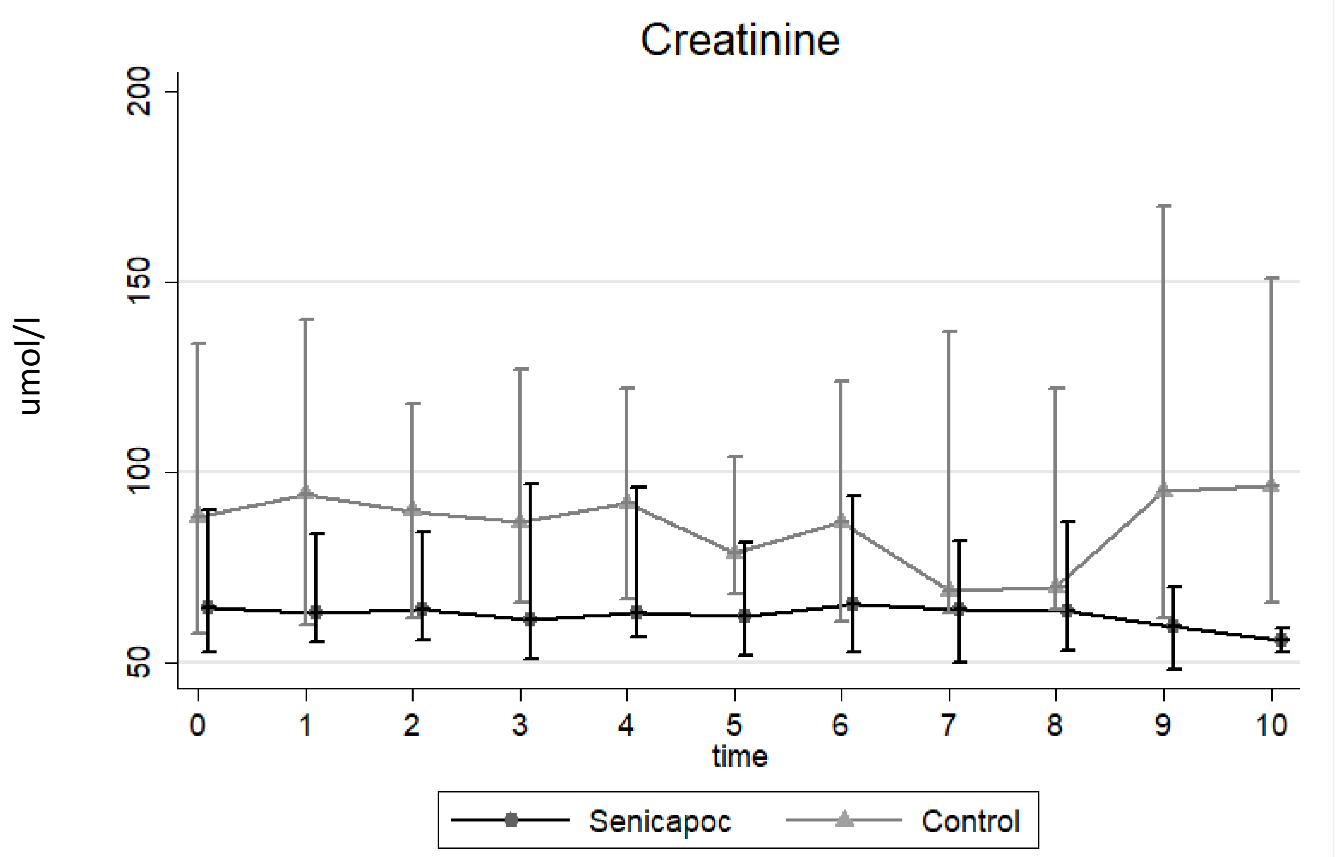

**No. of patients**											
**Control**	26	26	26	25	24	26	23	23	21	19	18
**Senicapoc**	20	20	20	20	18	20	14	17	16	16	13

Displayed as median (IQR)

eFigure 7 - Platelets

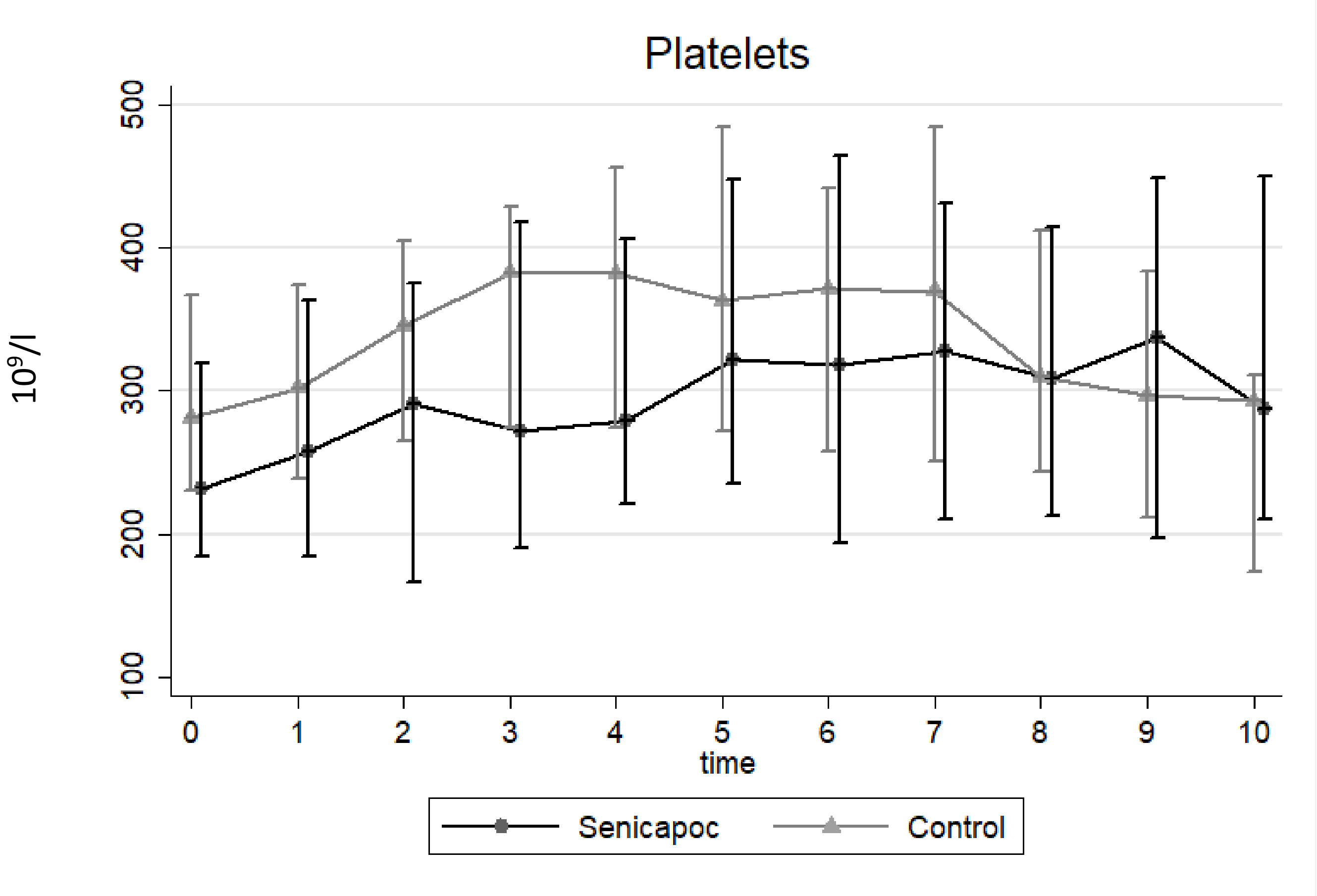

No. of patients											
**Control**	26	26	26	25	24	26	21	22	20	19	17
**Senicapoc**	20	20	20	20	17	18	15	15	17	15	13

Displayed as median (IQR)

eFigure 8 - Glukose

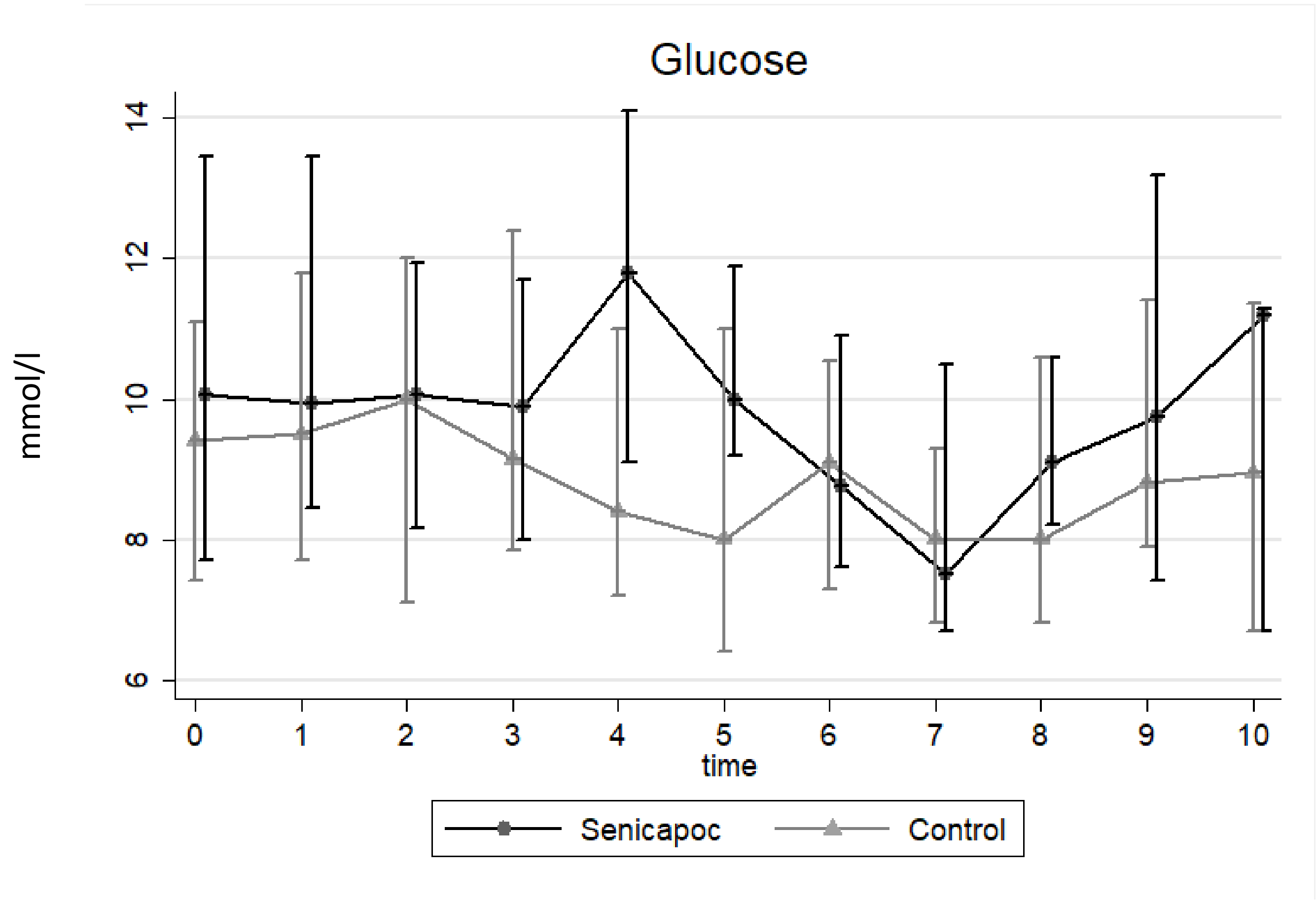

No. of patients											
**Control**	26	25	23	24	23	20	16	17	17	15	12
**Senicapoc**	20	20	20	19	15	13	12	11	10	10	9

Displayed as median (IQR)

eFigure 9 Virus Load

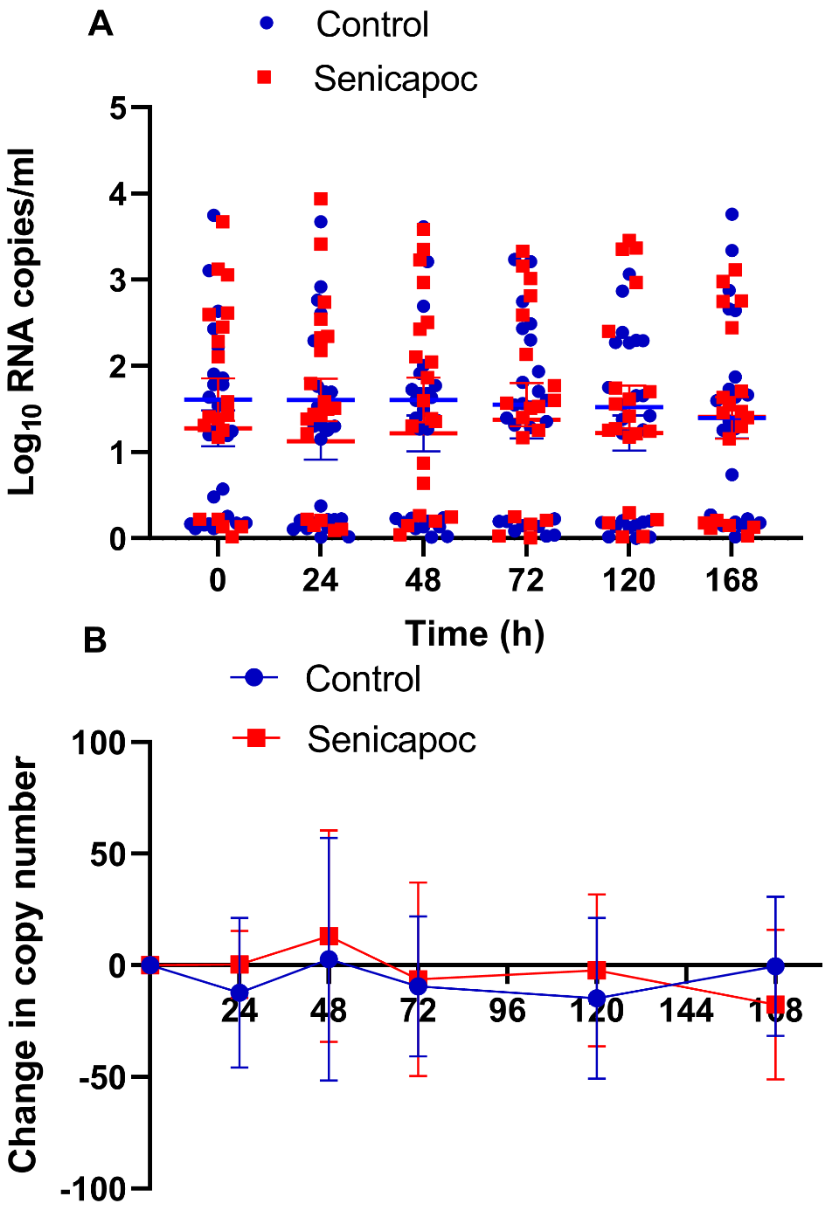

No. of patients						
**Time (hours)**	**0**	**24**	**48**	**72**	**120**	**168**
**Control**	25	26	25	24	25	21
**Senicapoc**	20	20	20	19	20	19

Number of SARS-CoV2 viral copies over time in absolute number (A) and relative to baseline (B)

Detection of SARS-CoV2 in plasma

Plasma samples (150 µl) from severely ill COVID-19 patient and supernatant from HEK2 cells were purified following the manufactures protocol, (NucleoSpin® Dx Virus kit, MACHEREY-NAGEL). Reverse transcription of 20 ul viral RNA was performed using SuperScript IV enzyme (ThermoFisher Scientific, Waltham, MA, USA), and incubated according to manufacturer's instructions in a thermal cycler (XT 96, VWR, Denmark).

QPCR was carried out with 2 µl of cDNA in a thermal cycler containing 0.25 mmol/l 1'-deoxynucleoside 5'- 224 triphosphate mix, 0.4 µmol/l forward primer and 1.2 µmol/l reverse primer, 1 x Ex Taq buffer and 0.2 U/µl TaKaRa Ex Taq Hot start DNA polymerase (TaKaRa Bio Europe, Göteborg, Sweden).

"Hot-start" procedure was employed for 1.5 minutes at 95 °C. Subsequent thermal cycling conditions were as follows: 15 seconds at 95 °C (denaturing) and 30 seconds at 58 °C (annealing) for 50 cycles.

Standard curves of viral RNA was performed using supernatant from HEK2 cells overexpressing ACE2 infected with SARS-Cov-2. The standard curves were used to estimate the relative virus-load of each patient sample. Negative controls were performed using plasma from blood samples from control persons without any symptoms and with negative swap PCR tests made from Statens Serum Institute, Denmark. The QPCR procedures were optimized to approach PCR efficiencies of 1. The results are presented as copies corresponding to the standard curve.

Primers and probes were designed using CLC Main Workbench (Qiagen, Redwood City, CA, USA) and synthesized at Eurofins Genomics, Ebersberg, Germany. The sequences are presented in the supplemental material (Table xx). Severe acute respiratory syndrome coronavirus 2, Wuhan-Hu-1 with accession no. NC_045512 was used as template of the primer design. The template primers used has originally been designed at Pasteur Institute, Paris for the RdRp gene and at the Charité protocol (1) for the E gene as a confirmatory assay with small changes in nucleotides sequence to optimize the protocol.

Table xx

Name	Gene	Sequence
IP2_12669-F	RdRp gene / nCoV_IP2	5´ATGAGCTTAGTCCTGTTG´3
IP2_12759-R	RdRp gene / nCoV_IP2	5´CTCCCTTTGTTGTGTTGT´3
IP2_12696-P	RdRp gene / nCoV_IP2	Hex 5´AGATGTCTTGTGCTGCCGGTA´3 BHQ1
IP4_14059-F	RdRp gene / nCoV_IP4	5´GGTAACTGGTATGATTTCG´3
IP4_14146-R	RdRp gene / nCoV_IP4	5´CTGGTCAAGGTTAATATAGG´3
IP4_14084-P	RdRp gene / nCoV_IP4	Fam 5´TCATACAAACCACGCCAGG´3 BHQ1
E_Sarbeco-F1	E gene / E_Sarbeco	5´ACAGGTACGTTAATAGTTAATAGCGT´3
E_Sarbeco-R2	E gene / E_Sarbeco	5´TACAAGACTCACGTTAACAATATTGCA´3
E_Sarbeco-P	E gene / E_Sarbeco	Fam 5´ACACTAGCCATCCTTACTGCGCTTCG´3 BHQ1

Reference

1. Corman VM, Landt O, Kaiser M, et al. Detection of 2019 novel coronavirus (2019-nCoV) by real-time RT-PCR. Euro Surveill 2020;25, 2000045. doi: 10.2807/1560-7917.ES.2020.25.3.2000045.

Fraction of inspired oxygen (FiO2) conversion tables

Nasal cannula: flow of oxygen and corresponding **FiO_2**

0 L/min:	**0.21**
1 L/min:	**0.27**
2 L/min:	**0.33**
3 L/min:	**0.37**
4 L/min:	**0.40**
5 L/min:	**0.44**
6 L/min:	**0.48**
10 L/min:	**0.62**

Hudson masks or similar:
Flow of oxygen and corresponding **FiO_2**

6 L/min:	**0.45**
8 L/min:	**0.50**
10 L/min:	**0.54**
15 L/min:	**0.59**
30 L/min:	**0.65**

Hudson mask or similar, when using **air/oxygen mixtures:**
Flow of oxygen/air and corresponding **FiO_2**

3 L O_2 / 12 L air /min (≈ 37%):	**0.29**
7.5 L O_2 / 7.5 L air /min (≈ 60%):	**0.41**
10 L O_2 / 5 L air /min (≈ 74%):	**0.48**
12 L O_2 / 18 L air /min (≈ 52%):	**0.39**

If a **Venturi-mask** is used, use the **FiO_2** as stated on the respective mask (color code), typical range **0.24** to **0.60**

If **high flow humidified oxygen via nasal cannula ≥ 15 L/min** is used:
The FiO_2 equals the oxygen concentration as stated on the mixer **(0.21 to 1.00)**

Reservoir-masks (non-rebreather masks) with flows ≥ 10 L/min, **FiO_2 = 0.95**

Protocol

Senicapoc in COVID-19 Patients with Severe Respiratory Insufficiency – A Randomized, Open-Label, Phase II Trial

Short name: COVIPOC

TRIAL PROTOCOL

Version 1.7

September 22, 2020

EudraCT number: 2020-001420-34

Sponsor

Ulf Simonsen, MD, PhD, DMSc

Professor

Department of Biomedicine

Aarhus University

Høegh-Guldbergs Gade 10

8000 Aarhus C, Denmark

Email: us@biomed.au.dk

Phone: +4560202613

Principal Investigator

Asger Granfeldt, MD, PhD, DMSc

Associate Professor

Department of Intensive Care

Aarhus University Hospital

Palle Juul-Jensens Boulevard 99

8200 Aarhus N, Denmark

Email: granfeldt@clin.au.dk

Phone: +4529720155

Content

Preface....6
List of abbreviations....7
Overview....8
Trial flow chart....9
Steering committee....10
Trial sites....12
Pharmacy....12
1. BACKGROUND....18
  1.1 COVID-19....18
    1.1.1 Epidemiology and disease....18
    1.1.2 Drugs for COVID-19....18
  1.2 Acute respiratory distress syndrome (ARDS)....19
    1.2.1 Incidence and mortality....19
    1.2.2 Unmet drug need for ARDS....19
    1.2.3 Pathophysiology....19
    1.2.4. Role of KCa3.1 channels in ARDS....19
  1.3 Senicapoc (ICA-17043 (bis(4-fluorophenyl)phenylacetamide)....19
    1.3.1 Pharmacology....19
    1.3.2 Senicapoc in ARDS....20
    1.3.3 Other potential uses of senicapoc....20
    1.3.4 Senicapoc and adverse events in clinical trials....21
    1.3.5 Human pharmacokinetics of senicapoc....22
    1.3.6 Dose considerations for ARDS-like disease....22
    1.3.7 Interaction of senicapoc with other drugs....23
  1.4 Standard of care....24
2. TRIAL OBJECTIVES AND HYPOTHESES....25
3. TRIAL DESIGN....26
  3.1 Overview....26
  3.2 Allocation....26
  3.3 Intervention....26
    3.3.1 Senicapoc....26
    3.3.2 Procedures and study medication....26
  3.4 Blinding....27

3.5 Trial procedures 27
3.5.1 Overview 27
3.5.2 Clinical personnel 27
3.5.3 Blood sampling 27
3.5.4 Drug traceability measure 27
4. SETTING AND PATIENT POPULATION 27
4.1 Setting 27
4.2 Inclusion criteria 28
4.3 Exclusion criteria 28
4.4 Co-enrollment 29
5. OUTCOMES 29
5.1 Primary outcome 29
5.1.1 Definition 29
5.1.2 Rationale 29
5.2 Secondary clinical outcomes 30
5.2.1 Definitions 30
5.2.2 Rationale 30
5.3 Additional clinical outcomes 30
5.4 Laboratory outcomes 31
5.4.1 Measurement of SARS-CoV2 load 31
5.4.2 Measurements of plasma senicapoc 31
5.4.3 Additional blood 31
5.5 Harm 32
5.5.1 General consideration 32
5.5.2 Definitions 32
5.5.3 Specific adverse events 32
5.5.4 Timeline 33
5.5.5 Suspected unexpected serious adverse reaction (SUSAR) 33
5.5.6 Reporting 34
6. SAMPLE SIZE CALCULATION AND STATISTICAL ANALYSIS PLAN 34
6.1 Sample size calculation 34
6.2 Statistical analysis plan 34
6.2.1 General considerations 34
6.2.2 Analysis of outcomes 35

6.2.3 Missing data 35
6.2.4 Multiple comparisons 35
6.2.5 Statistical stopping criteria 36
7. DATA COLLECTION AND MANAGEMENT 36
7.1 Data collection process 36
7.2 Variables 36
7.2.1 Overview 36
7.2.2 Variables collected 37
7.3 Data quality and validity 39
7.4 Data storage and security 39
7.5 Data access 39
8. CLINICAL TREATMENT 39
9. ETHICAL CONSIDERATIONS 40
9.1 Clinical equipoise 40
9.1.1 Potential benefits 40
9.1.2 Potential harms 40
9.1.3 Risk/benefit ratio 40
9.2 Research in critical illness 40
9.2.1 General considerations 40
9.2.2 Danish regulations 40
9.2.3 Regulations in relation to the current trial 41
9.3 Procedures 42
9.3.1 Ethical review committee 42
9.3.2 Trial-specific procedures 42
9.3.3 Insurance 43
10. MONITORING 43
10.1 Good Clinical Practice monitoring 43
11. TIMELINE AND ENROLLMENT 43
11.1 Timeline 43
11.2 Feasibility 43
11.3 Enrollment 44
12. PUBLICATION PLAN 44
13. DATA SHARING 44
14. FUNDING 44

15. PATENT 45
16. TASKS AND RESPONSIBILITIES 45
References 46
Appendix 1. Adverse events reported in trials of senicapoc 50
Appendix 2. Drug labeling (Danish) 53
Appendix 3. Potential interactions with drugs metabolized by CYP3A4 54

Preface

The COVIPOC trial will be conducted according to this protocol. The trial will be conducted in accordance with all applicable national and international laws, regulations, and guidelines including the revised version of the Declaration of Helsinki[1], European regulations[2], and the international Good Clinical Practice guidelines[3]. The trial and this protocol are developed in accordance with the International Conference on Harmonization (ICH) guidelines[3-5] and the Standard Protocol Items: Recommendations for Interventional Trials (SPIRIT) statement[6,7]. The sponsor took initiative to perform this study. The sponsor and the principal investigator wrote the protocol with input from the steering committee. Any substantial changes or amendments to the protocol will be clearly documented and communicated to all relevant parties.

	22-09-2020		22-09-2020
Ulf Simonsen, Sponsor	Date:	Asger Granfeldt, Principal Investigator	Date:

List of abbreviations

ARDS	Acute respiratory distress syndrome
CONSORT	Consolidated Standards of Reporting Trials
COVID-19	Coronavirus disease, i.e. disease with SARS-CoV-2
ECG	Electrocardiogram
eGFR	Estimated glomerular filtration rate
FiO_2	Fraction of inspired oxygen
ICU	Intensive care unit
LC–MS-MS	Liquid chromatography–mass spectrometry
MERS	Middle East Respiratory Syndrome
PaO_2	Partial pressure of oxygen in arterial blood
PCR	Polymerase chain reaction
REDCap	Research Electronic Data Capture
RNA	Ribonucleic acid
SARS-CoV-2	Severe acute respiratory syndrome coronavirus 2
SOFA	Sequential Organ Failure Assessment
SUSAR	Suspected unexpected serious adverse reactions
TBD	To be determined

Overview

Registry and trial number	EudraCT number: 2020-001420-34
Date of registration	March 27, 2020
Funding	Danish Ministry for Higher Education and Science
Sponsor	Ulf Simonsen, Aarhus University
Principal investigator	Asger Granfeldt, Aarhus University Hospital
Title	Senicapoc in COVID-19 Patients with Severe Respiratory Insufficiency – A Randomized, Open-Label, Phase II Trial
Country of recruitment	Denmark
Condition studied	COVID-19 patients with severe respiratory insufficiency
Intervention	Senicapoc
Comparator	None
Inclusion criteria	1) COVID-19<br>2) Age ≥18 years<br>3) Respiratory insufficiency requiring supplemental oxygen (> 10 L/min or mechanical ventilation with an FiO2 ≥ 40%)<br>4) Intensive care unit admission
Main exclusion criteria	1) Severe heart failure<br>2) Severe renal insufficiency<br>3) Hemodynamic instability<br>4) More than 24 hours since intensive care unit admission
Study type	Interventional<br>Allocation: Randomized (1:1)<br>Intervention model: Parallel group<br>Masking: Open label
Date of first screening	28.04.2020
Target sample size	46
Recruitment status	Started 23th of april 2020 7 patients have been included
Primary outcome	PaO_2/FiO_2 ratio 72 hours after randomization
Secondary outcomes	Ventilator-free days<br>28-day mortality

Trial flow chart

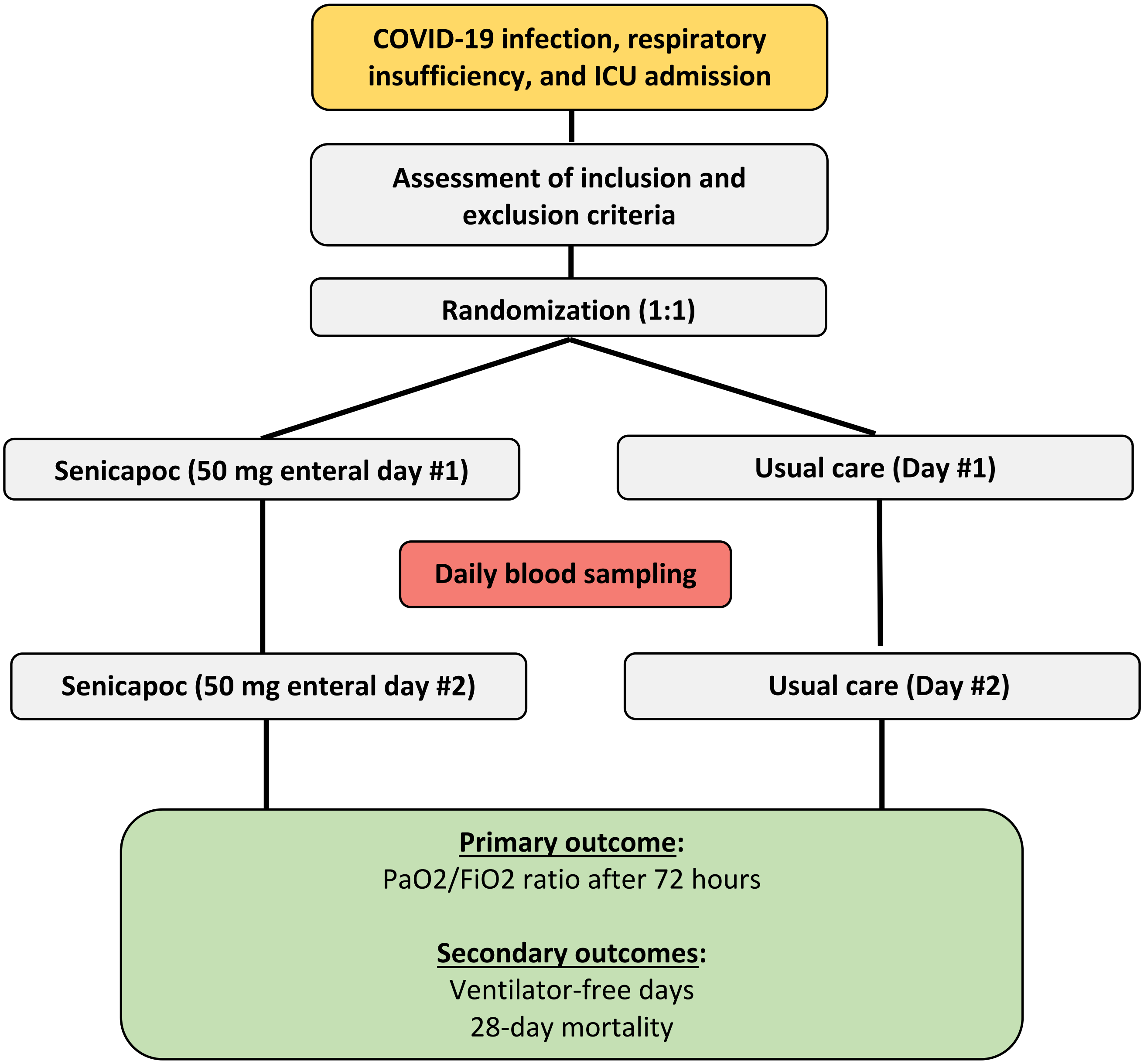

Steering committee

Ulf Simonsen, MD, PhD, DMSc
Sponsor
Professor
Department of Biomedicine
Aarhus University

Asger Granfeldt, MD, PhD, DMSc
Principal Investigator
Associate Professor
Department of Intensive Care
Aarhus University Hospital

Ole Hilberg, MD, DMSc
Professor
Department of Medicine
Vejle Hospital

Steffen Christensen, MD, PhD
Associate Professor
Department of Intensive Care
Aarhus University Hospital

Thomas Benfield, MD, DMSc
Professor
Department of Infectious Diseases
Hvidovre Hospital

Klaus Tjelle Kristiansen
Consultant
Department of Anesthesiology
Hvidovre Hospital

Isik Johansen, MD, DMSc
Professor
Department of Infectious Diseases
Odense University Hospital

Thomas Strøm, MD, PhD
Associate professor
Department of Anesthesiology
Odense University Hospital

Bodil S. Rasmussen, M.D., Ph.D.
Professor
Department of Anesthesiology and Intensive Care
Medicine, Aalborg University Hospital
Department of Clinical Medicine, Aalborg University

Olav Schjørring, M.D., Ph.D.
Department of Anesthesiology and Intensive Care
Medicine, Aalborg University Hospital
Department of Clinical Medicine, Aalborg University

Lars W. Andersen, MD, MPH, PhD, DMSc
Associate Professor
Research Center for Emergency Medicine
Aarhus University Hospital

Mikael Fink Vallentin, MD
Ph.D.-fellow
Prehospital Emergency Medical Services
Central Denmark Region
Department of Clinical Medicine
Aarhus University

Trial sites

Aarhus University Hospital

Palle Juul-Jensens Boulevard 99

8200 Aarhus N

Denmark

Site investigator: Steffen Christensen, e-mail: steffen.christensen@auh.rm.dk

Odense University Hospital

J. B. Winsløws Vej 4

5000 Odense C

Denmark

Site investigator: Thomas Strøm, e-mail: Thomas.Stroem@rsyd.dk

Hvidovre Hospital

Kettegård Alle 30

2650 Hvidovre

Denmark

Site investigator: Klaus Tjelle Kristiansen, e-mail: klaus.tjelle.kristiansen.02@regionh.dk.dk

Aalborg University Hospital – South

Hobrovej 18 – 22

9000 Aalborg

Denmark

Site investigator: Bodil Steen Rasmussen; bodil.steen.rasmussen@rn.dk

Pharmacy

Skanderborg Pharmacy

Adelgade 27

8660 Skanderborg

Denmark

Contact: Anna Marie Simonsen, ams@skanderborg-apotek.dk

Amendments

Version 1.6 to 1.7
The exclusion criteria has been changed from More than 12 hours since ICU admission to more than 24 hours since ICU admission

This has been changed as we have observed a delay in the time from taking the COVID-19 sample to an answer is provided.

Version 1.5 to 1.6
Aalborg University Hospital – has been added as a site.

Version 1.4 to 1.5
There has been changes in the primary investigator at Hvidovre
To

Klaus Tjelle Kristiansen
Consultant
Department of Anesthesiology
Hvidovre Hospital

From

Thomas Benfield, MD, DMSc
Professor
Department of Infectious Diseases
Hvidovre Hospital

Version 1.3 to 1.4

Section 7.2.1 Overview

Added the following statement

For patients included in the trial, name, unique patient identifier and inclusion/exclusion criteria will be stored in the trial database and is therefore disclosed to the investigators prior to obtaining consent.

Sections 5.5.3 Specific adverse events and 5.5.5 Suspected unexpected serious adverse reaction (SUSAR) has been updated.

Version 1.2 to 1.3

Added the following section

1.3.7 Interaction of senicapoc with other drugs

Added the following section 3.5.4 Drug traceability measure

Section 4.3 Exclusion criteria

Changed to

In fertile women (age < 60 years) a negative urine-hCG or plasma-hCG must be present before enrolment

Added

If documented in the medical record that the patient is infertile due to sterilization (hysterectomy, bilateral salpingectomy, or bilateral oophorectomy) testing for pregnancy is not needed.

Section 4.4 Co-enrollment

Added

Co-enrollment in other trials will be registered in the database.

In Section 5.3 Health-related quality of life (EQ-5D-5L86) at 28 days was added as an additional clinical outcome.

Sections 5.5.3 Specific adverse events and 5.5.5 Suspected unexpected serious adverse reaction (SUSAR) has been updated and expanded.

Section 7.2.1 Overview

Added

For patients not included in the trial, name, unique patient identifier and inclusion/exclusion criteria will be stored in the trial database and is therefore disclosed to the investigators

Drug labeling

Batchnummer: XXXX has been added to the drug label

Mikael Fink Vallentin, MD, Ph.D.-fellow has been added to the steering committee.

Version 1.1 to 1.2

Preface

Added:

The sponsor took initiative to perform this study.

Section 5.4.3 Additional blood

Changed from

The blood will be stored for a maximum of 5 years after which it will be destroyed

To

The blood will be stored in a research biobank for the purpose of this project only for a maximum of 5 years after which it will be destroyed

Section 7.2.1 Overview

Added

For patients not included in the trial, name, unique patient identifier and inclusion/exclusion criteria will be stored in the trial database.

Section 12. PUBLICATION PLAN

Changed from

Trial findings will be published irrespective of the results.

To

Trial findings will be published irrespective of whether the results are positive, neutral, negative or inconclusive.

Section 14. FUNDING.

Added

Each site will receive payment from the sponsor per included patient to cover expenses related to patient inclusion. This includes salaries and expenses related to blood sampling.

Version 1.0 to 1.1

Section 5.5.4 Timeline

Changed from

Adverse events will be collected until death or hospital discharge

Changed to

Adverse events will be collected until death, hospital discharge or a maximum of 28 days

There has been changes in the steering committee and as primary investigator at Odense University Hospital.

Palle Toft, MD, PhD, DMSc
Professor
Department of Anesthesiology
Odense University Hospital

Is replaced with

Thomas Strøm, MD, PhD
Associate professor
Department of Anesthesiology
Odense University Hospital

in the steering committee and as primary investigator at Odense University Hospital

The following has been added to the drug labeling

Nr. X (nummereres fortløbende)

1. BACKGROUND

1.1 COVID-19

1.1.1 Epidemiology and disease

COVID-19 is the disease caused by the severe acute respiratory syndrome coronavirus 2 (SARS-CoV-2). On March 12th 2020, the World Health Organization declared COVID-19 a global pandemic.[8] As of March 27th, 2020, more than 500.000 infected patients and 24.000 deaths have been reported with many more anticipated over the next months.[9]

COVID-19 is primarily characterized by upper or lower respiratory tract symptoms as well as fever.[10] The overall mortality from SARS-CoV-2 has been estimated at 1 to 6% with worse outcomes in patients suffering from chronic diseases such as respiratory disease, cardiovascular disease, hypertension, and diabetes.[10,11] Key markers implying a fatal outcome are acute respiratory distress syndrome (ARDS)-like disease with pronounced dyspnea, hypoxia, and radiological changes in the lung.[10,12,13] At this stage, disease progression is difficult to control despite current symptomatic treatment, including intensive care and respiratory support. Apart from symptomatic treatment, there is currently no pharmacological treatment with an effect on the condition, and the 28-day mortality might be a high as 50% in patients admitted to the intensive care unit (ICU) for ARDS-like disease in patients with COVID-19 infection.[13,14]

1.1.2 Drugs for COVID-19

The current approaches for the development of treatments for COVID-19 infection with ARDS-like disease range from the development of vaccines to small molecules targeting the virus receptor or intracellular replication of the SARS-CoV-2 virus, including testing of drugs for inhibition of human immunodeficiency virus. In brief, these approaches reduce the viral load, but currently, there is no evidence that they will lower the risk of ARDS emerging in the course of the SARS-CoV-2 infection.[15] The Ebola drug Remdesivir (Gilead) inhibits viral ribonucleic acid (RNA) polymerase in the Middle East Respiratory Syndrome (MERS), and currently at least 10 clinical trials administering the drug alone or in combination with other antiviral drugs are in progress. Camostat is a supposed inhibitor of the protease TMPRSS2 of importance for SARS-CoV-2 entry in the cell[16], and it is being tested in a clinical trial in Denmark. The antimalarial drug hydroxychloroquine was suggested to have effect on COVID-19 infection and more than 200 clinical trials are currently examining the effect of the drug on COVID-19 infection (ClinicalTrials.gov), but even using high clinical doses, hydroxychloroquine is only a weak inhibitor of KCa3.1 channels (Section 1.2.4).

1.2 Acute respiratory distress syndrome (ARDS)

1.2.1 Incidence and mortality

ARDS occurs in response to pulmonary and extra-pulmonary insults. The condition is characterized by disruption of the lung's alveolar-capillary membrane, leading to the development of pulmonary edema and severe hypoxemia.[17,18] Among ICU patients in general, approximately 10% have ARDS; and despite intensive supportive measures, the 28-day mortality remains high at approximately 40%.[19,20]

1.2.2 Unmet drug need for ARDS

There is currently no specific pharmacological treatments with an effect on ARDS.[21]

1.2.3 Pathophysiology

ARDS is associated with non-cardiogenic pulmonary edema because of an increased permeability for salts, proteins, and water in the lung.[17] Among the events leading to ARDS, is an immune cell mediated damage to the endothelial and epithelial barrier followed by leakage of protein-rich fluid into the interstitium and alveoli. The resulting alveolar flooding severely compromises gas diffusion which ultimately causes respiratory failure and hypoxia.[17]

1.2.4. Role of KCa3.1 channels in ARDS

We have described the expression of calcium-activated potassium channels with intermediate conductance (KCa3.1) in the lung epithelium and endothelial cells.[22] Following this observation, we discovered transgenic mice lacking KCa3.1 expression were protected against lung damage and accumulation of fluid in the lung.[23] In subsequent studies, we established an ventilator induced lung injury model representing the critical features of an ARDS-like disease. When KCa3.1 deficient mice or mice treated with senicapoc, a blocker of KCa3.1 channels, were subjected to the experimental protocol, we found that both groups were protected against lung injury (Petersen et al., submitted).

1.3 Senicapoc (ICA-17043 (bis(4-fluorophenyl)phenylacetamide)

1.3.1 Pharmacology

In preclinical studies, senicapoc is a selective and highly potent inhibitor of the KCa3.1 channels (the Gardos channel) with drug concentrations of 11 nM inhibiting 50% of K^+ efflux from red blood cells, by blocking the pore of the channel.[24] There is minimal effect of senicapoc on other K^+ and Na^+ channels, including those found in cardiac tissue, even when tested at concentrations 180-900 times greater than those used to inhibit the KCa3.1 channel.[25]

Senicapoc was developed for treatment of sickle cell anemia. Here, erythrocyte dehydration during sickling is predominantly caused by the potassium efflux through KCa3.1 channels.[26] In Phase-3 trials, testing was halted, as a treatment regime with senicapoc (20 mg twice daily for 4 days followed by a maintenance dose of senicapoc 10 mg once daily for the remainder of the treatment phase) failed to achieve its primary clinical end-point, a reduction in the incidence of painful vaso-occlusive crisis. This was despite clear improvements in hemoglobin levels and reductions of haemolysis.[27]

1.3.2 Senicapoc in ARDS

In the previously described animal study (Section 1.2.4), we found that that the KCa3.1 channel deficiency and the lowest applied dose of senicapoc (10 mg/kg) improved the primary outcome, the FiO_2/PaO_2 ratio. This suggest that treatment with senicapoc preserves lung function in ARDS-like disease.

Moreover, at higher doses (30-50 mg/kg) senicapoc treatment possesses anti-inflammatory effects illustrated as lower leukocyte accumulation inside the lungs after injury. Two other experimental animal studies support the anti-inflammatory role of KCa3.1 channel inhibition. First, in a mouse model of acute lung injury, KCa3.1 deficient mice were found to be significantly less effective at recruiting neutrophils into the site of inflammation.[28] Secondly, activation of KCa3.1 channels was found to mediate a release of interleukin 1β in macrophages, a mechanism blocked by the supratherapeutic concentrations of the weak inhibitor of KCa3.1 channels, hydroxychloroquine.[29]

In addition, there is evidence that senicapoc in high concentrations has antiviral properties and inhibits Arenaviruses[30], but currently it is uncertain whether senicapoc will have the same inhibitory effect on coronaviruses.

Based on our studies and the described pharmacology, we propose repurposing senicapoc for the treatment of ARDS-like disease, which has come highly relevant for treatment of COVID-19 infection.

1.3.3 Other potential uses of senicapoc

Senicapoc has been explored as a treatment for asthma in humans. A phase 2, randomized, double-blind, placebo-controlled, parallel-group, multicenter study with 34 patients was conducted to assess the safety and efficacy of two weeks of oral senicapoc administration (80 mg twice daily x 3 days followed by a maintenance dose of 40 mg daily for 11 days) on allergen challenge in atopic asthmatic subjects (NCT00861185, clinicaltrials.gov, sponsored by Icagen). In this trial, following two weeks of Senicapoc® treatment at 40 mg/day there was a reduction in the late allergen mediated increase in airway resistance along with a reduction in the inflammatory marker exhaled

nitric oxide. Senicapoc was also tested in 69 patients in a phase 2, randomized, double-blind, placebo-controlled, parallel-group, multicenter study to assess the safety and efficacy of four weeks of oral senicapoc administration on exercise-induced asthma (NCT00861211, clinicaltrials.gov, sponsored by Icagen). The senicapoc dosing was 80 mg twice daily for 3 days followed by a maintenance dose of 40 mg daily for remainder of the treatment period (total 4 weeks). In this trial, senicapoc's effect on exercise induced asthma was examined, with no improvement in lung function observed following 4 weeks of treatment.[31]

In two studies in a sheep model for pulmonary fibrosis, senicapoc was found to attenuate microvascular remodeling and lung fibrosis.[32,33]

In several animal models of neuroinflammation, senicapoc was found to have effect by blocking the KCa3.1 channels in astrocytes.[34] A clinical trial is planned with senicapoc 10 mg daily in patients with Alzheimer's disease (principal investigator Prof. John Olichney, UC Davies, USA).

1.3.4 Senicapoc and adverse events in clinical trials

Three senicapoc studies in healthy human volunteers have been conducted under a United States investigational new drug application. In these studies, 19 volunteers received placebo, and 68 received a single oral dose of senicapoc 25–200 mg.[35] Additional clinical trials of senicapoc in humans include a dose-escalating study[25], a phase II study[36], and a phase III study[27] for sickle cell disease, and two phase II studies for asthma (NCT00861185 and NCT00861211). The public available information on senicapoc and treatment-related adverse effects is from the phase I-III studies in patients with sickle cell anemia. This data is summarized below and provided tabulated in Appendix 1.

In a phase 1 dose-escalating trial in 21 patients, treated with up to 150 mg senicapoc or placebo, no important between-group differences were noted in occurrence of treatment-emergent adverse events except for nausea which seemed more frequent in the senicapoc 150-mg cohort.[25]

In the phase II trial, where placebo was compared to senicapoc 6 mg and 10 mg, all 90 patients were included in the safety analysis. Ten patients were prematurely discontinued from the study, 5 of whom were in the placebo group. Three of the 10 patients dropped out due to adverse events: 1 in the low-dose treatment group for weakness attributable to senicapoc. Five patients on placebo experienced at least one serious adverse event during dosing, while 7 patients in the low-dose arm and 6 patients in the high-dose arm experienced at least one serious adverse event. There were no notable changes in vital signs, physical examinations, ophthalmologic examinations, or electrocardiograms during the 12-week treatment period. Of particular importance, there were no differences in corrected QT interval when either of the senicapoc treatment groups was compared with the placebo group. The median glutamyl transferase value was increased in both the high-dose arm (10 U/L) and low-dose arm (2 U/L) compared with the placebo arm (0.2

U/L). There were no notable changes in any other laboratory tests monitored during the study. Finally, no meaningful differences were noted between treatment groups for any adverse events for those patients who did not enter the open-label extension but returned for a final study visit 8 weeks after the conclusion of dosing with trial medication.[27]

In the phase III study, patients with sickle cell disease were dosed senicapoc (n = 146) or placebo (n = 143). Events that occurred more frequently in the senicapoc versus placebo group (>5% difference) were nausea (16% vs. 10%) and urinary tract infections (14% vs. 8%). Nine patients (6%) in the placebo group and 12 patients (8%) in the senicapoc group dropped out of the study because of an adverse event. Sickle cell crisis-related events were the most common reason for discontinuation (one patient in the placebo and four patients in the senicapoc groups). Eighty-six patients (43/143, 30%, in the placebo group and 43/146, 29%, in the senicapoc group) experienced at least one serious adverse event other than sickle cell crisis. Pneumonia, catheter-related infections, anemia, fever, and asthma, were the most common serious events in both treatment groups and accounted for 81% (70/86) of the events, collectively. Two deaths considered unrelated to study treatment occurred in the placebo group during follow-up assessments. There were no clinically relevant changes in vital signs or physical examination findings between groups, and no differences between treatment groups or cohorts for any electrocardiogram (ECG) parameter, including heart rate, PR interval, QRS duration, or corrected QT interval. [36]

1.3.5 Human pharmacokinetics of senicapoc

After single dose peroral administration, the senicapoc plasma C_{max} increased (i.e., from 59.1 to 108.7 ng/ml) as the dose was increased from 50 to 100 mg. By contrast, little change in C_{max} was observed when the dose was increased from 100 to 150 mg (mean C_{max} increased only from 108.7 to 109.1 ng/ml). This finding suggests that in the capsule formulation of senicapoc, peak absorption of the drug plateaued at a single oral dose of approximately 100 mg. Median T_{max} across the three dose cohorts (50, 100, and 150 mg) appeared to be similar at 4, 6, and 4 hours, respectively.[25] The average terminal half-life of senicapoc in patients with sickle cell disease was 370 hours (15.4 days), 219 hours (9.1 days), and 297 hours (12.4 days) after single doses of 50, 100, and 150 mg, respectively.[25]

1.3.6 Dose considerations for ARDS-like disease

In the phase I study, a 50 mg dose administered peroral results in a C_{max} of 59.1 ng/ml (corresponding to 0.1828 µM). With a $t_{1/2}$ of 370 hours corresponding to k_e = 0.00187 hours^{-1}, the concentration after 24 hours will be 59.1 ng/ml * $e^{-0.00187}$ hours^{-1} * 24 hours = 56.5 ng/ml (corresponding to 0.1747 µM). The second dose of 50 mg will result in a C_{max} on day 2 of 115.5 ng/ml (corresponding to 0.3572 µM).

The concentration of senicapoc causing half-maximal inhibition of the KCa3.1 channels in human erythrocytes in vitro is 10 nM (corresponding to 3.23 ng/ml). Therefore, the plasma concentration of senicapoc with a dose regimen with 50 mg dosing at day 1 and day 2 will be above the ICC50 for senicapoc for 80 days (3.23 ng/ml = 115.5 ng/ml * $e^{-0.00187}$ $hours^{-1}$ * t, t = 1913 hours). Looking at the pharmacokinetic-pharmacodynamic relationship this will lead to inhibition of the KCa3.1 channels in the lung barrier and effect on the immune cells. The antiviral effect has only been measured in vitro and measurements of viral loads in the clinical trial will reveal whether it also contribute to the effect of senicapoc.

1.3.7 Interaction of senicapoc with other drugs

The potential for senicapoc to inhibit different cytochrome P450 systems has been studied in an in vitro study of seven recombinant human liver isozymes. At 10 µM, senicapoc was a poor inhibitor of human cytochrome P450 enzymes. Additionally, in vitro studies indicated no evidence of senicapoc metabolism by human hepatocytes. As such, senicapoc is expected to have a low propensity for drug-drug interactions that result from inhibition of metabolism.

Cytochrome P450 3A4 (CYP3A4) is a dominant drug-metabolizing enzyme and can be induced by commonly used drugs including dexamethasone, phenobarbital, rifampicin, and 1α,25-dihydroxyvitamin D3-[37,38] At concentrations as high as 1500 ng/mL (10-15 times Cmax estimated in section 1.3.5), senicapoc caused a 3.5-fold increase in CYP3A4 activity, which is below 5.8-fold, 13.4-fold, 9.8-fold, or 95.0-fold induction of CYP3A4 expression relative to that in the untreated controls observed with treatment with, respectively, dexamethasone, phenobarbital, rifampicin, and 1a,25-dihydroxyvitamin D3[39]. We therefore do not believe this is of clinical relevance.

In healthy volunteers, senicapoc at a 10 mg daily maintenance dose, increased CYP3A4 activity by 30%. In a separate study of healthy volunteers receiving a 20 mg daily maintenance dose there was a 20% increase in CYP3A4 activity. In a study of women receiving low dose birth control pills, concomitant administration of senicapoc caused a statistically significant increase (34% to 37%) in the urinary 6β-hydroxycortisol/cortisol ratio, indicating a modest induction of the cytochrome P450 isoenzyme 3A4 without altering serum progesterone and the ovulation remained suppressed, suggesting clinical significance is limited (Investigators Brochure). Patients admitted to an intensive care unit will not receive birth control pills and this is therefore not of clinical relevance. We will therefor register relevant drugs thought to be metabolized (substrates) by the CYP3A4 pathway in REDCap (see Appendix 3). The metabolism of senicapoc by liver enzymes in man is small, and it is unlikely that inhibitors of CYP3A4 will increase the plasma concentrations of senicapoc.

1.4 Standard of care

Currently there are no pharmacological treatment that can improve survival from ARDS.[21,40] The mainstay treatment of ARDS is therefore supportive with mechanical ventilation with low tidal volumes and low plateau pressures and accompanying sedation. Other supportive measures include vasopressor support, antibiotics, negative fluid balance, and renal replacement therapy when needed. In patient with severe ARDS prone positioning is also recommended. In patients with ARDS caused by COVID-19 treatment recommendations follows guidelines for the treatment of ARDS with emphasis on lung protective mechanical ventilation, high positive end expiratory pressures, negative fluid balance, empiric antimicrobial/antibacterial agents and prone positioning for patients with moderate to severe ARDS.[41]

2. TRIAL OBJECTIVES AND HYPOTHESES

Primary objective: To determine if administration of enteral Senicapoc improve the PaO_2/FiO_2 ratio after 72-hours in ICU patients with COVID-19 and respiratory insufficiency.

Primary hypothesis: We hypothesize that administration of enteral Senicapoc will improve the PaO_2/FiO_2 ratio at 72-hours in ICU patients with COVID-19 and respiratory insufficiency.

Secondary objective: To preliminary estimate the effect size of enteral Senicapoc in ICU patients with COVID-19 and respiratory insufficiency in regards to ventilator-free days and 28-day mortality in order to design a phase III trial.

3. TRIAL DESIGN

3.1 Overview

The COVIPOC trial is an investigator-initiated, randomized, open-label, phase II trial of senicapoc administration in ICU patients with COVID-19 and respiratory insufficiency. The trial will be conducted at three sites in Denmark and 46 patients will be included. The primary outcome is the PaO_2/FiO_2 ratio 72 hours after randomization. Key secondary clinical outcomes include ventilatory-free days and 28-day mortality.

3.2 Allocation

Patients will be randomized in a 1:1 ratio to either senicapoc + standard of care or standard of care alone in blocks with random sizes of 2 or 4. The randomization will be stratified according to the baseline PaO_2/FiO_2 ratio (above or below 150 mmHg [20 kPa]) and site. The randomized allocation list will be created by an independent statistician using a random number generator.

3.3 Intervention

3.3.1 Senicapoc

The intervention will consist of 50 mg enteral senicapoc administered as soon as possible after randomization and again after 24 hours. No further doses will be administered, and the study has no other interventions. Senicapoc is manufactured at Wuxi Apptec, San Diego, CA, USA and shipped to Denmark. The drugs will be QP released, packed, labeled and stored according to all relevant guidelines and regulations. This will be done by Skanderborg Pharmacy, a company that specializes in the production of medicine and is approved by the Danish Health authorities.

3.3.2 Procedures and study medication

Skanderborg Pharmacy will ship the drugs to participating sites prior to trial commencement and stored safely at room temperature under dry conditions, away from light. The drug will be delivered as 10 mg tablets and labelled according to Danish law (Appendix 2). Once the patient has been randomized to the senicapoc group, a nurse will dose five tablets (i.e. a total of 50 mg) and bring it to the patient. The drug will be administered by mouth (in awake patients) or by nasogastric tube if in place.

When a patient is randomized, an automatic notification will be sent to the site investigator and the principal investigator. This will ensure real-time tracking of available and used study medications.

3.4 Blinding

The trial will be open-label.

3.5 Trial procedures

3.5.1 Overview

The trial procedures will be limited to the intervention (Section 3.3), blood sampling (Section 3.5.3) and a phone call at 28 days to assess vital status if needed. Data will be obtained through review of electronic medical records. Patients will be included between 7 am and 22 pm seven days a week.

3.5.2 Clinical personnel

Prior to the beginning of patient enrollment and continuously throughout the enrollment period, the clinical teams involved in ICU care at the participating sites will be informed about the trial. This includes the trial's background, objectives, the inclusion/exclusion criteria, and the intervention.

3.5.3 Blood sampling

Blood sampling will be performed at the time of randomization prior to administration of the first study dose. Additional blood samples will be obtained after 24, 48, 72, 120, and 168 hours. 4 ml of venous or arterial blood will be collected in ethylenediaminetetraacetic acid tubes at each time point.

3.5.4 Drug traceability measure

Only one batch of senicapoc will used in the trial.

After being packed and labeled by Skanderborg Pharmacy the drugs are shipped to participating sites. When receiving the drugs this will be registered at each site. When a patient is randomized to senicapoc the study ID will be written on the pill bottle, while the unique number on the pill bottle label will be entered into REDCaAp. This will ensure that the drug can be traced from Skanderborg Pharmacy to the patient by to Skanderborg Pharmacy.

4. SETTING AND PATIENT POPULATION

4.1 Setting

The trial will be conducted in the ICUs at three Danish sites: Aarhus University Hospital, Odense University Hospital, and Hvidovre Hospital.

4.2 Inclusion criteria

Inclusion criteria:

1) COVID-19

2) Age ≥18 years

3) Respiratory insufficiency

4) ICU admission

COVID-19 will be defined as a positive polymerase chain reaction (PCR) test for SARS-CoV-2, either from a nasal or throat swab or from tracheal suctioning, within the last 14 days prior to ICU admission. Respiratory insufficiency will be defined as a need for supplemental oxygen of at least 10 L/min in patients without a need for mechanical ventilation or invasive or non-invasive mechanical ventilation with a $FiO_2 \geq 40\%$. An ICU will be defined per local practices.

4.3 Exclusion criteria

Exclusion criteria:

1) Severe heart failure (ejection fraction < 30%)

2) Severe renal insufficiency (eGFR < 30 mL/min/1.73m^2)

3) Severe hemodynamic instability (noradrenalin dose > 0.3 µg/kg/min)

4) Prior enrollment in the trial

5) Pregnancy

6) Allergy to senicapoc

7) Inability to take enteral medication

8) More than 24 hours since ICU admission

9) Limitations of care

10) Anticipated death within 24 hours

Severe heart failure will pragmatically be defined solely on the basis of the latest measured or estimated ejection fraction obtained via echocardiography (or other image modalities). If no ejection fraction is available, it will be assumed that the patient does not have severe heart failure. Renal insufficiency will be defined based on the latest estimated glomerular filtration rate (eGFR). If a patient is receiving renal replacement therapy, this will likewise be considered severe renal insufficiency. Hemodynamic instability will be defined based on a need for high-dose continuous vasopressor therapy specifically a noradrenalin dose > 0.3 µg/kg/min. If the patient is receiving other vasopressors instead of or in addition to noradrenalin, a

noradrenaline-equivalent dose will be estimated based on prior formulas.[42] In fertile women (age < 60 years) a negative urine-hCG or plasma-hCG must be present before enrolment. If it is documented in the medical record that the patient is infertile due to sterilization (e.g. hysterectomy, bilateral salpingectomy, or bilateral oophorectomy) testing for pregnancy is not needed. In order to optimize the chances of patients surviving to 72 hours (the time of the primary outcome), patients with limitations of care (including limitations related to mechanical ventilation and renal replacement therapy but not cardiopulmonary resuscitation) and patients with anticipated death within 24 hours, as judged by the treating clinician, will be excluded.

4.4 Co-enrollment

There will be no general restrictions on entry into other clinical trials although this will be evaluated on a case-by-case basis. Co-enrollment in other trials will be registered in the database.

5. OUTCOMES

5.1 Primary outcome

5.1.1 Definition

The primary outcome will be the PaO_2/FiO_2 ratio 72 hours after randomization. The PaO_2/FiO_2 ratio will be calculated based on the arterial gas closest to the time-point of 72 hours after randomization. If no clinically-indicated arterial gas is performed or planned within ± 2 hours of the time-point, an arterial gas will be performed. The ratio will be calculated based on the PaO_2 from the arterial gas and the FiO_2 at the same time point. If the patient is receiving mechanical ventilation (invasive or non-invasive) or is receiving oxygen through a high-flow nasal cannula, the FiO_2 will be obtained directly from the ventilator. If the patient is receiving oxygen through a regular nasal cannula, the FiO_2 will be calculated based on the following: FiO_2 = 21% + 4%/(l/min) x O_2 flow in l/min. For face masks, with or without reservoir, the FiO_2 will be estimated based on the approach used in EPIC2.[43,44] Patients who dies prior to the 72-hour time point will be handled as described in Section 6.2.3.

5.1.2 Rationale

The PaO_2/FiO_2 ratio is a commonly used measure of illness severity in patients with acute lung injury and ARDS and is used to define these two conditions.[17] Furthermore, the PaO_2/FiO_2 ratio is associated with mortality[17,20] making it a potential useful surrogate outcome for phase II trials. Although data is sparse, a lower PaO_2/FiO_2 ratio has also been associated with worse outcomes in patients with COVID-19.[10,45] Furthermore, animal studies in mice have shown that Senicapoc improves the PaO_2/FiO_2 ratio in experimentally induced ARDS (Section 1.3.2). Based on these considerations, and the fact that hypoxemic

respiratory failure is the hallmark of severe COVID-19 infection, the PaO_2/FiO_2 ratio is a reasonable primary outcome for a phase II trial. The primary measure of the PaO_2/FiO_2 ratio will be done 72-hours after randomization. This time-point was chosen to allow adequate time for the intervention to work while avoiding missing data due to deaths.

5.2 Secondary clinical outcomes

5.2.1 Definitions

The two key secondary clinical outcomes will be ventilator-free days and 28-day mortality.

Ventilator-free days will be defined as the number of days (or proportion of days) within the first 28 days after randomization where the patient is alive and not on invasive mechanical ventilation. Invasive ventilation is defined as mechanical ventilation through an endotracheal or tracheostomy tube. Whether or not a patient is receiving mechanical ventilation will be assessed on an hourly basis. If the patient dies within 28 days, the number of ventilator-free days will be zero.

5.2.2 Rationale

Ventilator-free days is a commonly used outcome, incorporating both freedom from ventilation and mortality, in trials of ARDS.[46,47] Assessment of mortality is considered a core outcome for trials within acute respiratory failure.[48] Both these outcomes are included to evaluate the relevance and feasibility of a future phase III trial.

5.3 Additional clinical outcomes

To assess the potential effects of the intervention on hemodynamics, we will measure vasopressor-free days. An infusion of a vasopressor will be defined as any continuous infusion of noradrenaline, dopamine, dobutamine, terlipressin, vasopressin, phenylephrine, and/or adrenaline. Vasopressor-free days will be defined as the number of days (or proportion of days) within the first 28 days after randomization where the patient is alive and not receiving vasopressors. Whether or not a patient is receiving vasopressors will be assessed on an hourly basis. If the patient dies within 28 days, the number of vasopressors-free days will be zero

To assess organ failure, we will calculate the Sequential Organ Failure Assessment (SOFA)-score[49] at 24, 48, 72, and 120 hours after randomization in those still alive. The SOFA score is a validated and widely used measure of organ failure assessing the respiratory, nervous, cardiovascular, hepatic, coagulation, and renal systems.[49] We will assess both the sub scores as well as the overall SOFA score. The calculation of the SOFA score will be based on available clinical and laboratory data. Laboratory and clinical data closest to the given

time point will be used. If a given component (e.g. bilirubin) is not available it will be assumed to be within normal ranges.

Need for renal replacement therapy within 28 days after randomization will be collected. Renal replacement therapy includes dialysis (hemodialysis or peritoneal dialysis), hemofiltration, and hemodiafiltration.

Health-related quality of life (EQ-5D-5L[50]) at 28 days will be assessed via telephone communication with the patient or a surrogate. The telephone interview will be semi-structured and based on the EQ-5D-5L questionnaire. The interview will be conducted by a centrally-located and trained member of the research team according to detailed standard operating procedures. In case the patient is still in the hospital, this interview will be face-to-face.

5.4 Laboratory outcomes

Blood samples are obtained to measure SARS-CoV-2 viral load and the concentration of Senicapoc.

5.4.1 Measurement of SARS-CoV2 load

Prior to randomization and after 72 hours blood is sampled, and virus is heat inactivated. The viral load is quantified by qPCR following standard protocol. A plasmid with part of the viral sequence is used as internal standard.

5.4.2 Measurements of plasma senicapoc

A high-performance liquid chromatography–mass spectrometry (LC–MS-MS) method to quantitate the level of senicapoc will be used.[25] Plasma samples will be diluted with an internal standard (ICA-18756, 2,2-bis-(4-fluoro-phenyl)-2-(2-fluoro-phenyl)-acetamide), and a 20-µl aliquot will be injected onto the LC-MS-MS system using cohesive turbulent flow technology. The validated method is established using 200 µL of plasma. The standard curve for ICA-17043 ranged from 1–300 ng/ml, and the lower limit of quantitation is established at 1 ng/ml.

5.4.3 Additional blood

Blood not used for analyses described in Section 5.4.1 and 5.4.2 will be stored at - 80°C for potential future analyses related to the current trial. The blood will be stored for a maximum of 5 years after which it will be destroyed. The blood will be stored in a research biobank for the purpose of this project only for a maximum of 5 years after which it will be destroyed

5.5 Harm

5.5.1 General consideration

Patients admitted to the ICU with COVID-19, and patients with ARDS in general, have a very high mortality.[10,13,20,45,51] Furthermore, these patients are at a high risk of organ failure and worsening of their clinical condition. Given this, it is impossible to comprehensively report all adverse events and assess their possible relationship with the intervention.

5.5.2 Definitions

The following definitions will be used:[2]

Adverse event: Any untoward medical occurrence in a subject to whom a medicinal product is administered and which does not necessarily have a causal relationship with this treatment

Serious adverse event: Any untoward medical occurrence that at any dose requires inpatient hospitalization or prolongation of existing hospitalization, results in persistent or significant disability or incapacity, results in a congenital anomaly or birth defect, is life-threatening, or results in death

Unexpected serious adverse reaction: A serious adverse reaction, the nature, severity or outcome of which is not consistent with the reference safety information

The reference document will be the investigator brochure for senicapoc (edition 9, Jan. 5 2009).

5.5.3 Specific adverse events

To assess specific adverse and potentially serious adverse events, in addition to that already collected (Section 7.2.2), we will collect data on the following

- Cardiac arrhythmias defined as an arrhythmia requiring pharmacological or mechanical intervention (direct current conversion or electrophysiological intervention).
- Vasopressor refractory shock defined as the need for noradrenalin infusion > 0.5ug/kg/min or equivalent of other vasopressors (noradrenaline, dopamine, dobutamine, terlipressin, vasopressin, phenylephrine, and/or adrenaline.) The noradrenaline-equivalent dose will be estimated based on prior formulas.[42]

- Allergic reaction defined as a systemic reaction involving symptoms from several organs including generalized urticaria.[52]
- Clinical diagnosis of acute coronary syndrome defined as new ischemic ECG changes, imaging evidence of new loss of viable myocardium or new regional wall motion abnormality in a pattern consistent with an ischemic etiology and identification of a coronary thrombus by angiography[53]
- Anemia defined as a hemoglobin level < 4.3 mmol/l
- Leucopenia defined as leukocyte count < 3.5 x 10^9 L^{-1}
- Severe hyperglycemia (blood glucose > 20 mmol/L)

Data on specific adverse events will be entered prospectively into REDCap which will ensure reporting to the sponsor. An e-mail will be send to the principal investigator when specific adverse events are entered into REDCap. The principal investigator will contact the site investigator and will together assess whether it is believed that the specific adverse events is associated with the investigational medicinal product or related to a general worsening of the patient's clinical condition. The investigators will inform the sponsor within 24 hours. If believed to be related to the investigational medicinal product it will be reported as a suspected unexpected serious adverse reaction (SUSAR) to the regulatory authorities immediately. If no assessment of causality is available the sponsor will contact the investigators. If the sponsor and the investigators disagree, it will be reported as a SUSAR and the report to the regulatory authorities will contain both statements.

5.5.4 Timeline

Adverse events will be collected until death, hospital discharge or a maximum of 28 days.

5.5.5 Suspected unexpected serious adverse reaction (SUSAR)

SUSARs will be reported immediately to the regulatory authorities. Given the consideration that patients admitted to the intensive care unit have a very high mortality and are at a high risk of organ failure and worsening of their clinical condition a very high of number of serious adverse events are expected. Serious adverse events are reported immediately to the site investigator and the principal investigator. The investigators will then assess whether it is believed that the serious adverse event is associated with the investigational medicinal product or related to a general worsening of the patient's clinical condition. The investigators will inform the sponsor within 24 hours.If believed to be related to the investigational medicinal product it will be reported as a SUSAR to the regulatory authorities immediately.. If no assessment

of causality is available the sponsor will contact the investigators. If the sponsor and the investigators disagree, the report to the regulatory authorities will contain both statements. This approach is compatible with ongoing Danish trials in critical ill patients (e.g. EudraCT number: 2017-004773-13 and 2019-003387-46). SUSARs will be reported to all site investigators within 24 hours.

5.5.6 Reporting

Once a year, or more often as appropriate, the sponsor will submit a list of all registered adverse events that have occurred during the trial period as well as a report on safety of the trial subjects to the Danish Medicines Agency and the National Committee on Health Research Ethics. The sponsor will notify both agencies when the trial has been completed (no later than 90 days thereafter) or if earlier than planned, the reasons for stopping the trial will be given. The results from the trial including important adverse events will be recorded on EudraCT.

6. SAMPLE SIZE CALCULATION AND STATISTICAL ANALYSIS PLAN

6.1 Sample size calculation

The sample size will be based on the primary outcome of the PaO_2/FiO_2 ratio at 72 hours. Given the novelty of COVID-19, there is limited data to support a definitive sample size calculation. Based on limited preliminary data[10,13,45], we expect that the PaO_2/FiO_2 ratio will be low signifying severe hypoxemic respiratory failure. We therefore anticipate that the PaO_2/FiO_2 ratio will be 120 mmHg (16 kPa) in the control group and 180 mmHg (24 kPa) in the Senicapoc group. With a common standard deviation of 70 mmHg, an alpha of 5%, and based on a t-test, 46 patients are needed to have 80% power to detect a statistically significant difference between groups. Power will further be increased by adjusting for the baseline PaO_2/FiO_2 ratio (Section 6.2.2).

The trial will not be powered to other outcomes.

6.2 Statistical analysis plan

6.2.1 General considerations

The statistical analyses and reporting will adhere to the Consolidated Standards of Reporting Trials (CONSORT)-guidelines.[54,55] All tests will be two-sided, a p-value <0.05 will be considered significant, and all confidence intervals will have 95% coverage.

In general, analyses will be conducted on an intention-to-treat basis including all randomized patients.

The two groups will be compared in relation to baseline characteristics using descriptive statistics.

6.2.2 Analysis of outcomes

The primary outcome, the PaO_2/FiO_2 ratio at 72 hours, will be compared between groups using linear regression adjusting for the two stratification variables (baseline PaO_2/FiO_2 ratio and site) as fixed effects. Results will be presented as a mean difference with 95% confidence intervals. If the outcome is substantially right-skewed, log-transformation will be considered. If this is necessary, results will be presented as a ratio of geometric means with 95% confidence intervals. If the model fails to converge or it is impossible to obtain approximately normally distributed outcome data, groups will be compared using the van Elteren test (a stratified extension of the Wilcoxon Rank Sum test)[56] with no direct estimate of the effect size. A similar approach will be used for other continuous outcomes.

Binary outcomes (e.g. mortality) will be compared between groups with logistic regression adjusting for the two stratification variables (baseline PaO_2/FiO_2 ratio and site) as fixed effects. Results will be presented as odds ratios with 95% confidence intervals.

6.2.3 Missing data

Missing data will be reported in relevant publications. We do not expect any missing data for the primary outcome except for patients dying prior to the 72-hour timepoint. For the primary analysis, we will use the last-observation carried forward approach and impute the last known PaO_2/FiO_2 ratio prior to the 72-hour time-point. We will conduct a number of sensitivity analyses to test the robustness of this approach. First, we will only analyze those who had a measured PaO_2/FiO_2 ratio at 72 hours. Second, for those without a PaO_2/FiO_2 ratio at 72 hours, we will impute the worst PaO_2/FiO_2 ratio at 72 hours observed in the entire population. Lastly, if feasible, multiple imputation will be performed taking into account prior PaO_2/FiO_2 ratios and the treatment group.

We do not expect missing data for the clinical key secondary outcomes. If missing data is > 5%, multiple imputation will be considered.

6.2.4 Multiple comparisons

No adjustments will be made for multiple comparisons. The rationale for this approach is three-fold. First, the trial has a clearly defined primary outcome which will ensure that the risk of a Type I error (i.e. false positives) is equal to the set alpha (i.e. 0.05) for this outcome. Second, the simplest procedure to control the family-wise error rate is the Bonferroni correction where the alpha is divided by the number of tests performed within the “family” of tests. However, defining the “family” is difficult and at best arbitrary.[57,58] Third, any adjustment for multiple comparisons to control the family-wise error rate increases the chance of Type II errors (i.e. false negatives).[58]

Given that the risk of Type I errors is not well defined when conducting multiple secondary analyses, these specific analyses should be considered exploratory and hypothesis generating.

6.2.5 Statistical stopping criteria

Since the primary outcome is not mortality, there will be no formal stopping criteria for efficacy. There will be no predefined stopping criteria for futility given the relatively small sample size. There will be no interim analyses.

7. DATA COLLECTION AND MANAGEMENT

7.1 Data collection process

Trained members of the research team, along with the site investigators, will be responsible for data collection and entry. All relevant data will be obtained from the electronical medical records, from bedside observation, analysis of obtained blood samples, or from the 28-day phone call.

7.2 Variables

7.2.1 Overview

All confirmed COVID-19 patients admitted to the ICU will be entered into a screening log. COVID-19 patients will be identified by review of all ICU admissions. Once a COVID-19 patient is identified, the electronic medical record will be reviewed for assessment of inclusion and exclusion criteria. For those not included in the trial, a specific reason for non-inclusion/exclusion will be documented. For patients not included in the trial, name, unique patient identifier and inclusion/exclusion criteria will be stored in the trial database, and is therefore disclosed to the investigators. For patients included in the trial, name, unique patient identifier and inclusion/exclusion criteria will be stored in the trial database and is therefore disclosed to the investigators prior to obtaining consent. All randomized patients will be entered into the main database.

A data dictionary that clearly defines all included variables will be created prior to patient enrollment. The data dictionary will provide the name of the variable (including the code used in the database), a detailed definition of the variable, categories for categorical variables, and units and ranges for continuous variables.

The number of collected variables will be kept relatively small to limit resource use and data entry mistakes. Below is provided a brief overview of the included variables, but details are reserved for the data dictionary.

7.2.2 Variables collected

Patient demographics and characteristics

- Name
- Unique patient identifier (Danish Central Personal Register number)
- Age
- Sex
- Height
- Weight

Conditions prior to the admission

- Co-morbidities
  - Cardiac
  - Non-cardiac
- Clinical fragility index

COVID-19 characteristics

- Symptoms
- Date of symptom onset
- Type and date of positive SARS-CoV-2 test

Admission and severity of illness at time of randomization

- Date and time of hospital admission
- Date and time of ICU admission
- SOFA score
- PaO_2/FiO_2 ratio
- Temperature
- Type of oxygen/ventilatory support including ventilatory settings
- Vasopressor use
- Sedation and paralytics
- Laboratory values
- Imaging findings

Treatments prior to randomization

- Antibiotics
- Corticosteroids
- Antivirals (e.g. Tamiflu, Remdesivir)
- Inclusion in other interventional trials

Trial related variables*

- Study ID
- Site
- Receipt of study drug
    - If no, reason for no study drug provided
- Administration route (oral or per nasogastric tube)
- Date and time of study drug administration
- Doses of study medication provided
- Inclusion criteria
- Exclusion criteria
- Date and time consent are obtained
- Time of blood sampling

*Trial related variables are entered directly into the electronic data base (i.e. there is no other source data)

Post-randomization variables

- PaO_2/FiO_2 ratio daily for the first 10 days
- SOFA score daily for the first 10 days
- Laboratory values daily for the first 10 days
- Type of oxygen/ventilatory support including ventilatory settings
- Sedation and paralytics
- Fluid balance
- Use of prone positioning
- Vasopressor use
- Renal replacement therapy
- Ventilator-free days
- Vasopressor-free days

- 28-mortality
- Health-related quality of life at 28 days

7.3 Data quality and validity

Data quality and validity will be optimized by having trained researchers enter all data according to a detailed data dictionary. REDCap (Section 7.4) is designed such that data forms contain field-specific validation checks ensuring that mandatory fields are filled out and that continuous variables are within predefined ranges. Given its limited utility, double-data entry will not be performed.[59,60]

7.4 Data storage and security

The database application we will use is REDCap.[61] REDCap is a professional database that provides a user-friendly interface. The REDCap data management system is secure, fully compliant with all regulatory guidelines, and includes a complete audit-trail for data entry validation. Through these mechanisms, as well as relevant training of all involved parties, patient confidentiality will be safeguarded.

Data will be handled according to all relevant Danish laws including the General Data Protection Regulation ("Databeskyttelsesforordningen") and the Data Protection Act ("Databeskyttelsesloven"). The project will be registered with the Central Denmark Region's internal list of research projects. Data will be stored for a minimum of 5 years.

7.5 Data access

During the trial, relevant members of the steering committee will have access to the entire database while site investigators will have access to local data only. The Good Clinical Practice unit, regulatory agencies, and other relevant monitoring entities will have direct access to patients' records and to all relevant trial data including all source data as applicable.

8. CLINICAL TREATMENT

The clinical management of included patients will be at the complete discretion of the treating clinicians in order to test the intervention in a real-life clinical scenario. In general, management will adhere to local, national, and international guidelines, but no specific treatments will be prohibited or mandated.

9. ETHICAL CONSIDERATIONS

9.1 Clinical equipoise

9.1.1 Potential benefits

Details about the potential benefits of the intervention are provided in the background section (Section 1.3.2). Based on the pharmacodynamic profile and animal data, there is compelling evidence that senicapoc could be beneficial in this condition.

9.1.2 Potential harms

Details about the potential harms of the intervention are provided in the background section (Section 1.3.4). As noted here, trials in humans have not found any serious adverse events associated with senicapoc. Additional potential harms related to the trial, e.g. blood sampling, are considered minimal.

9.1.3 Risk/benefit ratio

From the data provided above in Section 9.1.1. and 9.1.2 and in the background section, the current risk/benefit ratio is encouraging and there is therefore clinical equipoise for Senicapoc administration to patients with COVID-19 requiring ICU admission for respiratory insufficiency.

9.2 Research in critical illness

9.2.1 General considerations

Research in critical illness is ethically challenging for two reasons: 1) Patients are often unconsciousness or with reduced consciousness and can therefore not provide informed consent and 2) treatment must be administered within hours limiting the possibility of obtaining informed consent from a legally authorized representative. During pandemics, such as the current COVID-19 pandemic, where hospital visits from patients' next of kin is limited or prohibited, obtaining informed consent from a legally authorized representative is even more challenging. Despite these challenges, there is an ongoing need to conduct research in this, and similar, patient populations to improve outcomes. International guidelines, such as the revised Declaration of Helsinki[1], European regulations[2], and the Good Clinical Practice guidelines[3], clearly supports research in such populations.

The current trial will adhere to the revised Declaration of Helsinki as well as all applicable laws and regulatory guidelines.

9.2.2 Danish regulations

Danish law allows research without informed consent in situation where the following criteria are met:[62,63]

1) The research can only be conducted in the given acute situation
2) The patient is incapable of providing informed consent
3) Consent cannot be obtained from a surrogate given the urgency of the intervention
4) The research specifically involves the patient's current condition
5) There is a possibility of benefit to the patient

The current trial fulfils all the above criteria as described in Section 9.2.3 for #1-4 and in Section 9.1 for #5. Under these circumstances, research with pharmacological interventions is allowed if the following is obtained:[62-64]

1) Consent is obtained from a designated "legal guardian" ("forsøgsværge" in Danish)
2) Informed consent is obtained from the patient or a surrogate as soon as feasible

A "legal guardian" is a physician not involved in the research related to the specific patient and who is not in an inferior/superior position to the principal investigator or sponsor. The "legal guardian" should act according to the interest of the research participant.

9.2.3 Regulations in relation to the current trial

#1. The research can only be conducted in the given acute situation

Given the high morbidity and mortality of COVID-19 and ARDS, clinical trials are highly needed to improve patient outcomes. Animal studies do not adequately reflect the clinical condition and human trials are needed to advance treatment options. There is no other clinical condition that reflects this severe presentation of COVID-19, and any trial aimed to improve outcomes for COVID-19 patients with severe respiratory insufficiency can therefore only be conducted in this population.

#2. The patient is incapable of providing informed consent

Patients with severe respiratory insufficiency requiring admission to the ICU most often have reduced consciousness, are sedated, or are otherwise in an acute and critical state that does not allow for meaningful informed consent.[65-67]

#3. Consent cannot be obtained from a surrogate given the urgency of the intervention

COVID-19 induced respiratory insufficiency and ARDS more broadly are acute and severe conditions that can progress rapidly over a few hours. The intervention in the current trial is to be administered as soon as possible after admission to the ICU. Given these time frames, and the current restrictions on visits to the hospital, it would be impossible to obtain consent from a surrogate in a timely manner.

#4. The research specifically involves the patient's current condition
The interventions in this trial is specifically targeted for COVID-19 patients with respiratory insufficiency and if proven effective, will benefit this patient population.

9.3 Procedures

9.3.1 Ethical review committee
The trial will be sent for approval by the regional ethics committee.

9.3.2 Trial-specific procedures

The general procedures are consistent with other ongoing Danish trials in patients with acute and critical illness (EudraCT numbers: 2017-004773-13, 2019-003387-46, 2017-000632-34, and 2018-000404-42)

The "legal guardian" will be a physician not involved in trial procedures related to the specific patient. Consent for enrolment from the "legal guardian" will be obtained either through direct conversation or a phone call, while written consent will be obtained as soon as possible thereafter. The "legal guardians" will be informed of the trial including background, significance, inclusion- and exclusion criteria, as well as potential risks and benefits.

As soon as possible, a physician will obtain consent for further data collection from the patient or – if the patient is not able to provide consent – by a "legal guardian" and a surrogate. The physician obtaining consent will be a member of the steering committee or a physician with sufficient knowledge about the patient, the condition, and the trial (i.e. a member of the clinical team who has been informed about the trial and relevant procedures). Trial information and the consent request will take place in an undisturbed room if feasible, and the patient or the surrogate will have the opportunity to request an assessor. Between the trial information and the consent request, the patient or surrogate will be provided with an appropriate amount of time for consideration, and further time can be requested as needed.

The consent form will be signed by the patient or a surrogate, the person obtaining the consent, and the "legal guardian". If a patient dies before it is possible to obtain consent patient data will be included in the trial.[68] If a patient denies future participation in the trial, no additional data will be collected but all data collected up until the point of withdrawal will be included consistent with Danish law.[69]

When approached, the patient or a surrogate will be informed, verbally and in writing, about the background and significance of the study, inclusion criteria, potential risks and benefits, as well as a brief description of the study protocol. They will be informed that no additional interventions or procedures, except the 28-day telephone call (to determine survival status), will be performed and that future participation after the two drug doses will only include blood sampling and data collection. The patient or the surrogate will then provide written informed consent through the informed consent form approved by the ethical review committee. When consent is obtained from participants or a surrogate, information about potential deidentified data sharing will also be included.

The consent forms will be digital, and all signatures will be written on a smart phone or tablet using REDCap which has dedicated functionalities for written consent. This approach is consistent with an ongoing Danish trial (EudraCT numbers: 2019-003387-46).

9.3.3 Insurance

The patients in the study are covered by the Danish patient insurance.[70]

10. MONITORING

10.1 Good Clinical Practice monitoring

The trial will be monitored by the regional Good Clinical Practice monitoring unit. A detailed monitoring plan will be developed prior to trial commencement. The monitoring unit will have full access to all data in the trial.

11. TIMELINE AND ENROLLMENT

11.1 Timeline

Given the urgency of the COVID-19 pandemic, the aim is to start patient inclusion no later than April 15th 2020. We anticipate that enrollment will occur over a 2-month period with an additional month for patient follow-up. Trial results will be submitted for publication as soon as possible thereafter.

11.2 Feasibility

Based on current best estimates, a large number of COVID-19 patients will be admitted to the participating ICUs. Given the relatively broad inclusion and exclusion criteria, the majority of these patients will be eligible for the trial making enrollment of the required sample size highly feasible.

11.3 Enrollment

Enrollment at each site will be continuously monitored by the site investigator and the principal investigator. In case multiple eligible patients are not enrolled, a root cause analysis will be performed, and efforts will be made to avoid such issues in the future.

12. PUBLICATION PLAN

One main manuscript is planned from the current trial. The manuscript will adhere to the CONSORT guidelines[54,55] and will be published an in international peer-reviewed journal. The principal investigator will be the first author and the sponsor will be the last author. Additional authorship will follow authorship guidelines from the International Committee of Medical Journal Editors[71] and will include members of the steering committee and representatives from the sites as appropriate. Trial findings will be published irrespective of whether the results are positive, neutral, negative or inconclusive.

13. DATA SHARING

Six months after the publication of the results, all deidentified individual patient data will be made available for data sharing.[72] Procedures, including re-coding of key variables, will be put in place to allow for complete deidentification of the data. Data will be completely anonymized according to Danish law.

All relevant trial-related documents, including the protocol, data dictionary, and the main statistical code, will be made available for sharing along with the data. There will be no predetermined end date for the data sharing. Data will be available for any research purpose to all interested parties who have approval from an independent review committee and who have a methodological sound proposal as determined by the steering committee of the current trial. Only the methodological qualities and not the purpose or objective of the proposal will be considered. Interested parties will be able to request the data by contacting the principal investigator. Authorship of publications emerging from the shared data will follow standard authorship guidelines from the International Committee of Medical Journal Editors[71] and might or might not include authors from the steering committee depending on the nature of their involvement.

14. FUNDING

The project is supported by 5.3 mill. kr. from the Danish Ministry for Higher Education and Science. No one from the steering committee will receive honorary in connection with the clinical trial. Each site will receive payment from the sponsor per included patient to cover expenses related to patient inclusion. This includes salaries and expenses related to blood sampling.

15. PATENT

The investigator Ulf Simonsen, Ole Hilberg, and Asger Granfeldt have, through Aarhus University, patented senicapoc for use in the treatment of acute respiratory disease. In this case, respiratory disease is caused by an infection with a coronavirus (EP20163464, 2020). Aarhus University owns the patent and will receive the major benefit. According to Danish law a small part of eventual income by selling the patent will be given to the inventors.

16. TASKS AND RESPONSIBILITIES

<u>Principal investigator and sponsor</u>: Overall responsibility for protocol development, funding, budget overview, data dictionary development, ethical approval, trial registration, daily management, trial oversight, contact to the producer of the drug, contact to the Good Clinical Practice monitoring unit, assessment of overall recruitments, potential recruitment of additional sites, data analysis, and dissemination and presentation of results. Also, the responsibility to educate site investigators, evaluation of eligible patients not included, data entry and management, and patient follow-up.

<u>Steering committee</u>: Protocol development, funding, budget overview, data dictionary development, trial oversight, dissemination of results, responsibilities as principal investigator for short time periods.

<u>Site investigators and associated personnel</u>: Responsible for site-specific enrollment, evaluation of eligible patients not included, education of personnel at participating sites, reporting of site-specific issues or challenges to the principal investigator, participant consent for data collection.

References

1. World Medical Association. World Medical Association Declaration of Helsinki: ethical principles for medical research involving human subjects. *JAMA.* 2013;310(20):2191-2194.
2. The European Parliament and the Council of the European Unit. Clinical Trials Regulation (CTR) EU No 536/2014. 2014; https://ec.europa.eu/health/sites/health/files/files/eudralex/vol-1/reg_2014_536/reg_2014_536_en.pdf. Accessed July 18, 2017.
3. ICH Harmonised Tripartite Guideline. Integrated Addendum To ICH E6(R1): Guideline For Good Clinical Practice E6(R2). 2016; http://www.ich.org/fileadmin/Public_Web_Site/ICH_Products/Guidelines/Efficacy/E6/E6_R2__Step_4.pdf. Accessed June 30, 2017.
4. ICH Harmonised Tripartite Guideline. General Considerations for Clinical Trials E8. 1997; http://www.ich.org/fileadmin/Public_Web_Site/ICH_Products/Guidelines/Efficacy/E8/Step4/E8_Guideline.pdf. Accessed June 30, 2017.
5. ICH Harnomised Tripartite Guidelines. Statistical Principles for Clinical Trials. 1998; http://www.ich.org/fileadmin/Public_Web_Site/ICH_Products/Guidelines/Efficacy/E9/Step4/E9_Guideline.pdf. Accessed June 30, 2017.
6. Chan AW, Tetzlaff JM, Altman DG, et al. SPIRIT 2013 statement: defining standard protocol items for clinical trials. *Ann Intern Med.* 2013;158(3):200-207.
7. Chan AW, Tetzlaff JM, Gotzsche PC, et al. SPIRIT 2013 explanation and elaboration: guidance for protocols of clinical trials. *BMJ.* 2013;346:e7586.
8. Corvera JS, Kin H, Dobson GP, et al. Polarized arrest with warm or cold adenosine/lidocaine blood cardioplegia is equivalent to hypothermic potassium blood cardioplegia. *J Thorac Cardiov Sur.* 2005;129(3):599-606.
9. Cronstein BN, Kramer SB, Rosenstein ED, Weissmann G, Hirschhorn R. Adenosine modulates the generation of superoxide anion by stimulated human neutrophils via interaction with a specific cell surface receptor. *Ann N Y Acad Sci.* 1983;451:291-301.
10. Guan WJ, Ni ZY, Hu Y, et al. Clinical Characteristics of Coronavirus Disease 2019 in China. *N Engl J Med.* 2020.
11. Baud D, Qi X, Nielsen-Saines K, Musso D, Pomar L, Favre G. Real estimates of mortality following COVID-19 infection. *Lancet Infect Dis.* 2020.
12. Yoon SH, Lee KH, Kim JY, et al. Chest Radiographic and CT Findings of the 2019 Novel Coronavirus Disease (COVID-19): Analysis of Nine Patients Treated in Korea. *Korean J Radiol.* 2020;21(4):494-500.
13. Arentz M, Yim E, Klaff L, et al. Characteristics and Outcomes of 21 Critically Ill Patients With COVID-19 in Washington State. *JAMA.* 2020.
14. Huang C, Wang Y, Li X, et al. Clinical features of patients infected with 2019 novel coronavirus in Wuhan, China. *Lancet.* 2020;395(10223):497-506.
15. Cao B, Wang Y, Wen D, et al. A Trial of Lopinavir-Ritonavir in Adults Hospitalized with Severe Covid-19. *N Engl J Med.* 2020.
16. Hoffmann M, Kleine-Weber H, Schroeder S, et al. SARS-CoV-2 Cell Entry Depends on ACE2 and TMPRSS2 and Is Blocked by a Clinically Proven Protease Inhibitor. *Cell.* 2020.
17. Force ADT, Ranieri VM, Rubenfeld GD, et al. Acute respiratory distress syndrome: the Berlin Definition. *JAMA.* 2012;307(23):2526-2533.
18. Thompson BT, Chambers RC, Liu KD. Acute Respiratory Distress Syndrome. *N Engl J Med.* 2017;377(19):1904-1905.
19. Luhr OR, Antonsen K, Karlsson M, et al. Incidence and mortality after acute respiratory failure and acute respiratory distress syndrome in Sweden, Denmark, and Iceland. The ARF Study Group. *Am J Respir Crit Care Med.* 1999;159(6):1849-1861.

20. Bellani G, Laffey JG, Pham T, et al. Epidemiology, Patterns of Care, and Mortality for Patients With Acute Respiratory Distress Syndrome in Intensive Care Units in 50 Countries. *JAMA.* 2016;315(8):788-800.
21. Lewis SR, Pritchard MW, Thomas CM, Smith AF. Pharmacological agents for adults with acute respiratory distress syndrome. *Cochrane Database Syst Rev.* 2019;7:CD004477.
22. Kroigaard C, Dalsgaard T, Nielsen G, et al. Activation of endothelial and epithelial K(Ca) 2.3 calcium-activated potassium channels by NS309 relaxes human small pulmonary arteries and bronchioles. *Br J Pharmacol.* 2012;167(1):37-47.
23. Wandall-Frostholm C, Dalsgaard T, Bajoriunas V, et al. Genetic deficit of KCa 3.1 channels protects against pulmonary circulatory collapse induced by TRPV4 channel activation. *Br J Pharmacol.* 2015;172(18):4493-4505.
24. Nguyen HM, Singh V, Pressly B, Jenkins DP, Wulff H, Yarov-Yarovoy V. Structural Insights into the Atomistic Mechanisms of Action of Small Molecule Inhibitors Targeting the KCa3.1 Channel Pore. *Mol Pharmacol.* 2017;91(4):392-402.
25. Ataga KI, Orringer EP, Styles L, et al. Dose-escalation study of ICA-17043 in patients with sickle cell disease. *Pharmacotherapy.* 2006;26(11):1557-1564.
26. Bunn HF. Pathogenesis and treatment of sickle cell disease. *N Engl J Med.* 1997;337(11):762-769.
27. Ataga KI, Reid M, Ballas SK, et al. Improvements in haemolysis and indicators of erythrocyte survival do not correlate with acute vaso-occlusive crises in patients with sickle cell disease: a phase III randomized, placebo-controlled, double-blind study of the Gardos channel blocker senicapoc (ICA-17043). *Br J Haematol.* 2011;153(1):92-104.
28. Henriquez C, Riquelme TT, Vera D, et al. The calcium-activated potassium channel KCa3.1 plays a central role in the chemotactic response of mammalian neutrophils. *Acta Physiol (Oxf).* 2016;216(1):132-145.
29. Eugenia Schroeder M, Russo S, Costa C, et al. Pro-inflammatory Ca(++)-activated K(+) channels are inhibited by hydroxychloroquine. *Sci Rep.* 2017;7(1):1892.
30. Lawler PG, Hewlett AM. Clinical evaluation of the iso-shunt diagram [proceedings]. *Br J Anaesth.* 1978;50(1):77-78.
31. Wulff H, Castle NA. Therapeutic potential of KCa3.1 blockers: recent advances and promising trends. *Expert Rev Clin Pharmacol.* 2010;3(3):385-396.
32. Organ L, Bacci B, Koumoundouros E, et al. Inhibition of the KCa3.1 Channel Alleviates Established Pulmonary Fibrosis in a Large Animal Model. *Am J Respir Cell Mol Biol.* 2017;56(4):539-550.
33. Derseh HB, Dewage SNV, Perera KUE, et al. KCa3.1 channel blockade attenuates microvascular remodelling in a large animal model of bleomycin-induced pulmonary fibrosis. *Sci Rep.* 2019;9(1):19893.
34. Staal RGW, Weinstein JR, Nattini M, Cajina M, Chandresana G, Moller T. Senicapoc: Repurposing a Drug to Target Microglia KCa3.1 in Stroke. *Neurochem Res.* 2017;42(9):2639-2645.
35. Ataga KI, Stocker J. Senicapoc (ICA-17043): a potential therapy for the prevention and treatment of hemolysis-associated complications in sickle cell anemia. *Expert Opin Investig Drugs.* 2009;18(2):231-239.
36. Ataga KI, Smith WR, De Castro LM, et al. Efficacy and safety of the Gardos channel blocker, senicapoc (ICA-17043), in patients with sickle cell anemia. *Blood.* 2008;111(8):3991-3997.
37. van de Kerkhof EG, de Graaf IA, Ungell AL, Groothuis GM. Induction of metabolism and transport in human intestine: validation of precision-cut slices as a tool to study induction of drug metabolism in human intestine in vitro. *Drug Metab Dispos.* 2008;36(3):604-613.
38. Doherty MM, Charman WN. The mucosa of the small intestine: how clinically relevant as an organ of drug metabolism? *Clin Pharmacokinet.* 2002;41(4):235-253.
39. Negoro R, Takayama K, Nagamoto Y, Sakurai F, Tachibana M, Mizuguchi H. Modeling of drug-mediated CYP3A4 induction by using human iPS cell-derived enterocyte-like cells. *Biochem Biophys Res Commun.* 2016;472(4):631-636.

40. Fan E, Brodie D, Slutsky AS. Acute Respiratory Distress Syndrome: Advances in Diagnosis and Treatment. *JAMA.* 2018;319(7):698-710.
41. Murthy S, Gomersall CD, Fowler RA. Care for Critically Ill Patients With COVID-19. *JAMA.* 2020.
42. Brand DA, Patrick PA, Berger JT, et al. Intensity of Vasopressor Therapy for Septic Shock and the Risk of In-Hospital Death. *J Pain Symptom Manage.* 2017;53(5):938-943.
43. Vincent JL, Rello J, Marshall J, et al. International study of the prevalence and outcomes of infection in intensive care units. *JAMA.* 2009;302(21):2323-2329.
44. https://www.intensive.org/epic2/Documents/Estimation%20of%20PO2%20and%20FiO2.pdf.
45. Yang X, Yu Y, Xu J, et al. Clinical course and outcomes of critically ill patients with SARS-CoV-2 pneumonia in Wuhan, China: a single-centered, retrospective, observational study. *Lancet Respir Med.* 2020.
46. Schoenfeld DA, Bernard GR, ARDS Network. Statistical evaluation of ventilator-free days as an efficacy measure in clinical trials of treatments for acute respiratory distress syndrome. *Crit Care Med.* 2002;30(8):1772-1777.
47. Yehya N, Harhay MO, Curley MAQ, Schoenfeld DA, Reeder RW. Reappraisal of Ventilator-Free Days in Critical Care Research. *Am J Respir Crit Care Med.* 2019;200(7):828-836.
48. Needham DM, Sepulveda KA, Dinglas VD, et al. Core Outcome Measures for Clinical Research in Acute Respiratory Failure Survivors. An International Modified Delphi Consensus Study. *Am J Respir Crit Care Med.* 2017;196(9):1122-1130.
49. Vincent JL, Moreno R, Takala J, et al. The SOFA (Sepsis-related Organ Failure Assessment) score to describe organ dysfunction/failure. On behalf of the Working Group on Sepsis-Related Problems of the European Society of Intensive Care Medicine. *Intensive Care Med.* 1996;22(7):707-710.
50. Herdman M, Gudex C, Lloyd A, et al. Development and preliminary testing of the new five-level version of EQ-5D (EQ-5D-5L). *Qual Life Res.* 2011;20(10):1727-1736.
51. Thompson BT, Chambers RC, Liu KD. Acute Respiratory Distress Syndrome. *N Engl J Med.* 2017;377(6):562-572.
52. Castells M. Diagnosis and management of anaphylaxis in precision medicine. *J Allergy Clin Immunol.* 2017;140(2):321-333.
53. Thygesen K, Alpert JS, Jaffe AS, et al. Fourth universal definition of myocardial infarction (2018). *Eur Heart J.* 2019;40(3):237-269.
54. Schulz KF, Altman DG, Moher D, Group C. CONSORT 2010 statement: updated guidelines for reporting parallel group randomised trials. *BMJ.* 2010;340:c332.
55. Moher D, Hopewell S, Schulz KF, et al. CONSORT 2010 explanation and elaboration: updated guidelines for reporting parallel group randomised trials. *BMJ.* 2010;340:c869.
56. van Elteren P. On the Combination of Independent Two Sample Tests of Wilcoxon. *Bulletin of the Institute of International Statistics.* 1960;37:351-361.
57. Rothman KJ. No Adjustments Are Needed for Multiple Comparisons. *Epidemiology.* 1990;1(1):43-46.
58. Feise RJ. Do multiple outcome measures require p-value adjustment? *BMC Med Res Methodol.* 2002;2:8.
59. Gibson D, Harvey AJ, Everett V, Parmar MK. Is double data entry necessary? The CHART trials. CHART Steering Committee. Continuous, Hyperfractionated, Accelerated Radiotherapy. *Control Clin Trials.* 1994;15(6):482-488.
60. Day S, Fayers P, Harvey D. Double data entry: what value, what price? *Control Clin Trials.* 1998;19(1):15-24.
61. Harris PA, Taylor R, Thielke R, Payne J, Gonzalez N, Conde JG. Research electronic data capture (REDCap)--a metadata-driven methodology and workflow process for providing translational research informatics support. *J Biomed Inform.* 2009;42(2):377-381.
62. Lov om videnskabsetisk behandling af sundhedsvidenskabelige forskningsprojekter [Danish]. 2011; https://www.retsinformation.dk/forms/r0710.aspx?id=137674#Not1. Accessed July 7, 2017.

63. Vejledning om akutte forsøg [Danish]. 2017; http://www.nvk.dk/emner/akutte-forsoeg/vejledning-om-akutte-forsoeg#1. Accessed July 7, 2017.
64. Lov om kliniske forsøg med lægemidler [Danish]. 2016; https://www.retsinformation.dk/Forms/r0710.aspx?id=180117. Accessed July 7, 2017.
65. Truog RD. Informed consent and research design in critical care medicine. *Crit Care.* 1999;3(3):R29-R33.
66. Ecarnot F, Quenot JP, Besch G, Piton G. Ethical challenges involved in obtaining consent for research from patients hospitalized in the intensive care unit. *Ann Transl Med.* 2017;5(Suppl 4):S41.
67. Matei M, Lemaire F. Intensive care unit research and informed consent: still a conundrum. *Am J Respir Crit Care Med.* 2013;187(11):1164-1166.
68. Ministeriet for Sundhed og Forebyggelse. Letter to Den Nationale Videnskabsetiske Komité dated February 28th, 2014 regarding "Anvendelse af data i akutforsøg" [Danish].
69. Lov om ændring af lov om videnskabsetisk behandling af sundhedsvidenskabelige forskningsprojekter [Danish]. 2012; https://www.retsinformation.dk/forms/r0710.aspx?id=142351. Accessed July 13, 2017.
70. Bekendtgørelse af lov om klage- og erstatningsadgang inden for sundhedsvæsenet [Danish]. 2011; https://www.retsinformation.dk/Forms/r0710.aspx?id=138893. Accessed July 13, 2017.
71. International Committee of Medical Journal Editors. Defining the Role of Authors and Contributors. 2017; http://www.icmje.org/recommendations/browse/roles-and-responsibilities/defining-the-role-of-authors-and-contributors.html. Accessed July 6, 2017.
72. Taichman DB, Sahni P, Pinborg A, et al. Data Sharing Statements for Clinical Trials - A Requirement of the International Committee of Medical Journal Editors. *N Engl J Med.* 2017;376(23):2277-2279.

Appendix 1. Adverse events reported in trials of senicapoc

Phase I study in sickle-cell disease[25]

Single dose of senicapoc (50, 100, or 150 mg) or placebo

Adverse Event[a]	**Senicapoc** (n = 21) No. (%)	**Placebo** (n = 7) No. (%)
Sickle cell pain crisis	2 (10)	1 (14)
Nausea	4 (19)	1 (14)
Dry mouth or throat	2 (10)	0
Fatigue	2 (10)	0
Pyrexia or increased body temperature	1 (5)	2 (29)
Pharyngitis or nasopharyngitis	2 (10)	1 (14)
Dehydration	2 (10)	0
Arthralgia	2 (10)	1 (14)
Back pain	2 (10)	2 (29)
Myalgia	2 (10)	0
Headache	2 (10)	1 (14)
Dizziness	2 (10)	0
Somnolence	2 (10)	0
Pruritus	2 (10)	0

[a] Patients were counted only once for each body system and preferred term. Adverse events were included in the table if two or more patients reported an event in the two groups combined. Some events considered similar were grouped.

Phase II study in sickle-cell disease[36]

Incidence of most frequent adverse events[a]			
**Adverse event[a]**	**Placebo,** (n = 30) No. (%)	**6 mg senicapoc** (n = 29) No. (%)	**10 mg senicapoc** (n = 31) No. (%)
Diarrhea	1 (3)	2 (7)	5 (16)
Nausea	1 (3)	3 (10)	4 (13)
Constipation	1 (3)	4 (14)	0
Gastroenteritis	3 (10)	0	1 (3)
Upper respiratory tract infection	3 (10)	5 (17)	1 (3)
Chest pain	0	3 (10)	1 (3)
Increased SGOT	3 (10)	0	1 (3)
Arthralgia	4 (13)	2 (7)	3 (10)
Back pain	4 (13)	4 (14)	2 (6)

[a] Any event that was present in 10% or more of a given dosing group is listed.

Incidence of serious adverse events			
**Serious adverse event**	**Placebo,** (n = 30) No. (%)	**6 mg senicapoc** (n = 29) No. (%)	**10 mg senicapoc** (n = 31) No. (%)
Sickle cell crisis	5 (17)	5 (17)	5 (16)
Pneumonia	2 (7)	1 (3)	1 (3)
Acute chest syndrome	0	1 (3)	0
Staphylococcal sepsis	0	1 (3)	0
Urinary tract infection	0	0	1 (3)
Muscle strain	0	0	1 (3)
Aseptic necrosis of the bone	0	1 (3)	0
Bronchitis	1 (3)	0	0
Deep vein thrombosis	0	0	1 (3)

Phase III study in sickle-cell disease[27]

**Adverse events**	**Senicapoc** (n = 146) No. (%)	**Placebo** (n = 143) No. (%)
At least 1 event	127 (87)	119 (83)
Nausea	23 (16)	14 (10)
Urinary tract Infection	21 (14)	12 (8)
Headache	20 (14)	23 (16)
Arthralgia	13 (9)	7 (5)
Upper respiratory tract Infection	12 (8)	20 (14)
Vomiting	12 (8)	11 (8)
Pyrexia	12 (8)	15 (10)
Pneumonia	11 (8)	13 (9)
Back pain	11 (8)	10 (7)
Pain in extremity	11 (8)	9 (6)
Nasopharyngitis	10 (7)	11 (8)
Cough	10 (7)	6 (4)
Constipation	9 (6)	13 (9)
Fatigue	9 (6)	8 (6)
Hypokalemia	7 (5)	3 (2)
Hematuria	7 (5)	1 (<1)
Diarrhea	5 (3)	12 (8)
Abdominal pain	5 (3)	7 (5)
Pharyngolaryngeal pain	4 (3)	10 (7)
Pruritus	4 (3)	7 (5)
Drug hypersensitivity	2 (1)	7 (5)

Appendix 2. Drug labeling (Danish)

Kun til anvendelse i klinisk forsøg<br>**COVIPOC** EudraCT nr.: 2020-001420-34<br><br>Nr. X (nummereres fortløbende)<br><br>Batchnummer: XXXXX<br><br>Studie ID: __________<br>(udfyldes efter randomisering)<br><br>Senicapoc tabletter 10 mg, 10 stk.<br><br>Administreres peroralt eller i sonde.<br><br>Forsøgsdosis 50 mg<br><br>Opbevares ved stuetemperatur, tørt og uden direkte lys.<br><br>Udløb: XXXXX<br><br>Forespørgsler vedr. forsøget skal ske til:<br>Forsøgsansvarlig Asger Granfeldt<br>Aarhus Universitetshospital Skejby<br>Palle Juul-Jensens Boulevard 99,<br>8200 Aarhus N.<br>granfeldt@clin.au.dk<br>Mobilnr.: 29720155

Appendix 3. Potential interactions with drugs metabolized by CYP3A4

Table 1. Potential interactions with drugs metabolized by CYP3A4

**Drug class or type**<br>**Drug or compound**	**Interaction/effect**
Antibiotic, Anti-tuberculosis<br>- RIFAMPICIN	CYP3A4- inducer
Anti-convulsive<br>- CARBAMAZEPIN	CYP3A4- inducer (and substrate)
Anti-convulsive –<br>- PHENOBARBITAL<br>- PHENYTOIN	CYP3A4-inducer
Anti-emetics<br>- APREPITANT<br>- FOSAPREPITANT	CYP3A4-inhibitor (and substrates)
Anti-mycotics<br>- ITRACONAZOL<br>- KETOCONAZOL	CYP3A4-inhibitor (and substrates)
Anti-viral drugs<br>- HIV-PROTEASE-inhibitors, e.g., ritonavir, indinavir	CYP3A4-inhibitors (and substrates)
Calcium channel blockers<br>- DILTIAZEM	CYP3A4-inhibitor (and substrate)
HMG Co-A reductase inhibitors<br>- STATINS, e.g. simvastatin, atorvastatin	CYP3A4-inhibitor (and substrate)
Oral contraceptives	Senicapoc co-administration partially reverses the suppressive effect of the oral contraceptive on pituitary-ovarian function. However, ovulation remains suppressed with no effect on serum progesterone.
Immunosuppressive<br>- CICLOSPORIN	CYP3A4-inhibitor (and substrate)
Macrolide antibiotics<br>- CLARITHROMYCIN<br>- ERYTHROMYCIN	CYP3A4-inhibitor (and substrate)
Endogenous and synthetic corticosteroids<br>- DEXAMETHASONE	Upregulation of CYP3A4 may interfere with the glucocorticoid metabolism

In cases where the drug is a substrate for CYP3A4, senicapoc may increase the metabolism and lower the activity of the drug. There are no indications that senicapoc will increase concentration of other drugs.
